# The Dual Associations of Peripheral Inflammatory Cells With Brain Reorganization in Insular Gliomas With/Without Epilepsy: An Exploratory Analysis

**DOI:** 10.1002/cns.70788

**Published:** 2026-02-20

**Authors:** Hongfang Zhao, Bohan Zhang, Qifeng He, Zhenghai Deng, Zonggang Hou, Jian Xie

**Affiliations:** ^1^ Capital Medical University Beijing China; ^2^ Department of Neurosurgery, Beijing Tiantan Hospital Capital Medical University Beijing China; ^3^ Department of Neurology, Beijing Tiantan Hospital Capital Medical University Beijing China

**Keywords:** brain reorganization, epilepsy, insular glioma, morphological analysis, peripheral blood biomarker

## Abstract

**Objectives:**

The insula was invaded by gliomas and epilepsy occurred frequently. Our study aimed to reveal brain reorganization and explore potential peripheral biomarkers that reflect these processes.

**Methods:**

51 insular glioma‐related epilepsy (IRE) and 52 without epilepsy (IRnE) patients were included. Deep learning was used for tumor segmentation to generate masks for subsequent analyses. Virtual brain grafting was applied to eliminate the mass effect. Morphological analyses relied on MATLAB, SPM, and CAT12. Statistical analyses included χ^2^ test, *t* test, one‐way ANOVA, regression, principal component, and correlation analysis.

**Results:**

Increased gyrification (*GI*) occurred on the contralateral side of IRnE. Increased gray matter volume and toroidal *GI* (*Toro GI*) appeared on the same and contralateral sides of IRE, respectively. Elevated *Toro GI* showed an initial predictive value for the control of postoperative epilepsy and was negatively associated with blood white cell, monocyte, and neutrophil counts in IRE. The opposite relationship was observed for GI in IRnE.

**Significance:**

The brain would conduct different reorganization models to adapt to IRE and IRnE. These processes may be associated with specific peripheral inflammatory cells. Longitudinal studies and explorations of more specific biomarkers are necessary for elucidating the causal relationships and providing suggestions for therapeutic concepts of different insular glioma subtypes.

## Graphical abstract

Legend process. (A,B) Based on deep learning and virtual brain grafting, we revealed that the brain could conduct reorganization to adopt insular glioma with/without epilepsy, which was not influenced by tumor features and was associated with peripheral inflammatory cells. (C) Exploring causal links of the associations may provide personal suggestions for insular glioma.

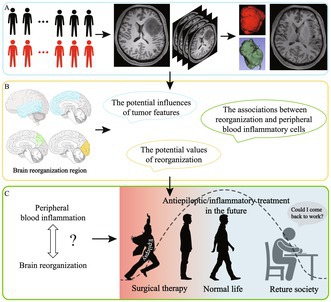

## 1 Introduction

Glioma was the common primary intracranial malignant tumor and involved the insular lobe frequently [1, 2]. Clinical experience demonstrated that insular glioma‐related epilepsy (IRE) would affect patients' lives severely [3]. With advanced high‐resolution multimodal MRI, researchers discovered some brain topological changes in glioma and seizure patients [4]. Based on graph theory analysis, Camins et al. (2022) and He et al. (2024) found that IRE could damage fronto‐occipital nerve fibers and brain functional networks [5, 6]. Additionally, Ciolac et al. (2022) and Xue et al. (2024) reported that gray matter volume and structural complexity in several regions would increase as the brain adapted to epilepsy [7, 8]. It meant that there was a subtle balance between damage and recombination in neural architecture [9]. However, the morphological analyses of IRE and IRnE (insular glioma without epilepsy) were fewer, due to the tumor mass effect [10, 11]. Previous studies shown that virtual brain grafting (VBG), as an open‐source workflow, was designed to mitigate the confounding influence of tumor occupancy effect in imaging analyses. Based on the corresponding tumor masks, it could offer generated eligible brain images for further studies by conducting robust processes for original structural MRI, such as imaging flipping, donor image generation, and lesion filling [12]. Its stability had been confirmed by current research [12, 13]. Additionally, with the further requirement of accurate masks and extracted features in radiomics, the deep learning and nnU‐Net_v2 model were widely utilized. Its core advantage was an automated and adaptive pipeline covering data preprocessing, network structure selection, training strategies, and inference procedures. It could provide relatively accurate tumor masks for VBG and subsequent processing. In our study, we aimed to explore the structural changes as the brain faced IRE and IRnE using this combined approach.

Additionally, recent studies supported that brain reorganization was associated with specific cerebrospinal fluid (CSF) cytokines in some diseases, such as multiple sclerosis and epilepsy [14, 15]. However, challenges in obtaining pre‐ or intra‐operative samples, along with tumor heterogeneity, have limited further exploration of these associations in IRE. Considering these difficulties, peripheral blood examination, as a non‐invasive approach, gained increasing attention. Previous studies revealed a substantial overlap of inflammatory and epileptic indicators among serum, CSF, and solid tissues [16]. Additionally, Su et al. (2025) indicated that peripheral metabolic products were negatively related to gray matter volume in some mental disorders [17]. These findings supported that peripheral blood components may reflect the process of brain recombination. In this exploratory study, we investigated brain reorganization after eliminating the mass effect and explored potential associations between reorganization and peripheral blood components. By bridging imaging with clinical information, we hope to provide clinicians with different aspects to understand IRE, offer suggestions for further identification of more specific biomarkers in CSF and solid tissues, and support new insights for future therapeutic strategies (Graphical Abstract).

## 2 Materials and Methods

### 2.1 Participants

The study was approved by the Institutional Review Board of Beijing Tiantan Hospital. Written informed consent was obtained from each participant (KY 2020–146‐02). The inclusion criteria were as follows: (i) diagnosis of insular glioma confirmed according to the WHO criteria; (ii) age ≥ 18 years; (iii) no prior biopsy, surgery, radiotherapy, or chemotherapy before MRI acquisition; (iv) absence of the significant midline shift caused by the mass effect; (v) mild to moderate brain edema without abnormal gray/white matter differentiation or surface structural distortion; and (vi) the types of epilepsy limited to generalized tonic–clonic seizures and absence seizures, which could be diagnosed based on clinical symptoms and electroencephalogram findings.

### 2.2 Clinical Information and MR Imaging Parameters

Patient information was obtained from medical records, including demographic data, preoperative laboratory results, histopathological, and molecular features. The tumor location, volume, and MR imaging parameters (Table S1) were derived from preoperative MRI data.

### 2.3 Image Preprocessing

#### 2.3.1 Glioma Segmentation

##### 2.3.1.1 Hardware and Environmental Setup

Glioma segmentation was performed using the deep learning and nnUNet_v2 model pipeline [18]. All segmentation tasks were conducted on a workstation running the Ubuntu 22.04 operating system (https://ubuntu.com/), equipped with a 14‐core CPU, 32 GB RAM, and an NVIDIA RTX 4090 GPU (24 GB VRAM). The software environment was built on Python 3.12 and PyTorch 2.3.0 with CUDA 12.1 support (https://www.python.org/; https://pytorch.org/; https://developer.nvidia.com/cuda‐toolkit). The nnUNet_v2 framework was installed relying on an Anaconda virtual environment (https://www.anaconda.com/).

##### 2.3.1.2 nnUNet_v2 Deployment Pipeline

The framework was deployed by cloning the official repository and performed an editable installation. Initially, we utilized the Task01_BrainTumor dataset from the Medical Segmentation Decathlon, whose reliability had been demonstrated in numerous competitions [19]. Subsequently, we conducted network deployment based on the official description, such as the working directory setup and environment variable configuration. Automatic model training included data format conversion, data fingerprinting, integrity verification, experimental designation, and data preprocessing. To ensure the segmentation accuracy, the 3D full‐resolution model, 200 training epochs, and five‐fold cross‐validation were selected for model identification. Finally, we identified the best model in automatic model optimization (Figure S1).

##### 2.3.1.3 Inference Process

According to the nnU‐Net_v2 requirements, multimodal sequences (T1, T2, T1c, and FLAIR) were input after the necessary preprocessing, including data conversion, spatial normalization, bias field correction, and skull stripping. All lesion masks generated by the model were reviewed by two board‐certified neuroradiologists independently. Manual delineation would be performed if the generated segmentation was not accurate.

#### 2.3.2 Brain Reconstruction

The brain reconstruction was performed using the VBG pipeline, T1‐weighted sequence data, and corresponding tumor masks. The pipeline automatically completed lesion filling through the following core procedures: (i) Basic preprocessing: image reorientation, denoising, bias field correction, skull stripping, and spatial registration. These steps ensured that the brain structure was accurately extracted from the T1‐weighted data and mapped to the standard template; (ii) initial donor image generation: imaging flipping, stitched brain, and initial filled brain. The VBG pipeline mirrored the brain and filled the tumor affected regions with normal brain structures based on tumor masks generated by nnUNet_v2; and (iii) final donor image generation was achieved through weighted blending and mathematical refinement [12].

#### 2.3.3 Morphometric Analysis

The processing relied on the MATLAB R2022b, SPM12 v7771, CAT12 v12.8.2 (www.mathworks.com/products/new_products/release2022b.html; www.github.com/spm/spm12; www.github.com/ChristianGaser/cat12) and the following steps: (i) Voxel‐based morphometry analysis (VBM) involved automated tissue segmentation, spatial normalization, the generation of gray matter/white matter and cerebrospinal fluid probability maps using the diffeomorphic anatomical registration through the DARTEL algorithm (Tissue Probability Maps: TPM.nii; Affine Regularization: East Asian brains; Space Template: MNI152.nii); (ii) surface‐based morphometry analysis (SBM) processing included cortical surface reconstruction, topology correction, spherical registration, and surface‐based normalization, resulting in the generation of central cortical surfaces for each hemisphere; (iii) morphological features were extracted from the central surfaces by CAT12, such as gray matter volume (GMV), fractal dimension (*FD*), gyrification (*GI*), toroidal GI (*Toro GI*), depth and thickness; (iv) spatial smoothing was applied using an 8 mm Gaussian kernel for GMV and a 12 mm kernel for surface‐related indices. J.X. (with more than 20 years of experience) evaluated the gray matter and surface mesh using the CAT12 quality reports and visual checks. Data were excluded if the quality did not meet the required threshold (B level) or if abnormalities were observed, such as voids in the surface mesh, extreme values in the cortical thickness map, surface fractures or discontinuities, or abnormal sulcal patterns (Figure S2).

### 2.4 Statistical Analysis

Clinical information was compared using the χ^2^ test, *t* test, and one‐way ANOVA. Morphological analyses were performed using MATLAB, SPM, and CAT12. Multiple comparisons were corrected using a family‐wise error rate. Subsequently, we extracted structural indices from significantly different regions and conducted post hoc comparisons using the Newman–Keuls multiple comparison test. To facilitate observation, we regarded some reorganization regions as different wholes based on the tumor side and the presence of epilepsy and performed principal component analyses (PCA) based on z‐score standardized variables on the structural indices [20]. Regression analyses were conducted to assess the predictive value of structural indices for postoperative seizure control and tumor recurrence. Linear regression analyses were used to explore the associations between clinical variables and brain reorganization. The significance threshold was set at *p* < 0.05 (two‐tailed).

## 3 Results

### 3.1 Descriptive Analysis and Preoperative Examination Analysis

Initially, 110 insular glioma patients and 50 healthy controls (HCs) were recruited in this study. Owing to low imaging quality or refusal to undergo surgery, 7 patients were excluded. Consequently, a total of 103 patients with insular glioma (51 with IRE) were included finally. The recruitment process and tumor overlap map are shown in Figure S3. Patients were classified according to age (> 40 years) and the Ki‐67 level (> 10%) based on previous studies [18, 21]. There was no significant difference among the IRE, IRnE, and HC groups in demographic or clinical information (Table 1). Regarding peripheral blood components, there were significant differences between IRE and IRnE. For instance, the counts of white blood cells (WBC), lymphocytes, and platelets were more elevated in IRnE. The percentage of monocytes was relatively decreased in IRnE. There was no difference in liver or renal function (Table 2).

**TABLE 1:** Demographic and clinical characteristics of healthy controls, IRE, and IRnE patients.

Factors		IRE	IRnE	HC	*p*
Gender (women/men)		51(20/31)	52(20/32)	51(26/25)	0.30
Age (year; Mean ± SD)		42.94 ± 12.16	43.15 ± 13.24	42.04 ± 11.07	0.68
Handedness (R/L)		54/0	52/0	51/0	N/A
Lateral (R/L)		26/25	27/25	N/A	0.92
Duration of illness (day, Median (IQR))		60 (25–300)	90 (30–195)		0.63
Tumor volume (cm^3^; Mean ± SD)		91.19 ± 58.54	95.46 ± 65.03		0.72
WHO grade	Low grade (grade II)^a^	26	23		0.42
	High grade (grade III‐IV)	25	29		
Histopathological subtype	Oligodendroglioma	14	10		0.12
	Astrocytoma	19	27		
	Anaplastic Oligodendroglioma	0	3		
	Anaplastic Astrocytoma	8	3		
	Glioblastoma	10	9		
IDH status (Mutation/Wild‐type)		30/21	38/14		0.13
1p/19q(co‐delete/non co‐delete)		14/37	13/39		0.91
MGMT (Methylation/Unmethylation/unidentified)		17/11/23	19/8/25		0.45
ATRX status (Mutation/Wild‐type/unidentified)		19/22/10	18/32/2		0.32
TP53 (Mutation/Wild‐type/unidentified)		20/22/9	24/26/2		0.97
TERT (Mutation/Wild‐type/unidentified)		13/14/24	9/26/27		0.08
Ki‐67 status (<10%/>10%)		27/24	26/26		0.77
Postoperative epilepsy (Y/N/missed)		7/34/10	6/38/8		0.66
Tumor recurrence (Y/N/missed)		9/32/10	11/32/9		0.70
Survival status (Y/N/missed)		6/35/10	4/39/9		0.45

Abbreviations: 1p/19q, 1p/19q Chromosome Codeletion; ATRX, Alpha Thalassemia/Mental Retardation Syndrome X‐linked; HC, health control; IDH, Isocitrate Dehydrogenase; IQR, interquartile range; IRE, insular glioma related epilepsy; IRnE, insular glioma without epilepsy; L, left; MGMT, O‐6 Methylguanine‐DNA Methyltransferase; N, No; *p*, *p* value; R, right; SD, standard deviation; TERT, Telomerase Reverse Transcriptase; TP53: Tumor Protein 53; Y, Yes.

^a^ There was no glioma patients diagnosed with WHO grade I because of its relatively less common among adults.

**TABLE 2:** Details about peripheral blood components in IRE and IRnE.

Characteristics	IRE (Mean ± SD)	IRnE (Mean ± SD)	χ^2^	*p*
WBC	6.28 ± 1.52	6.91 ± 1.67	2.00	0.05
RBC	4.69 ± 0.51	4.81 ± 0.44	1.36	0.18
NEUT	4.16 ± 1.78	4.56 ± 2.06	1.13	0.26
LY	1.78 ± 0.61	2.03 ± 0.61	2.13	0.04
MONO	0.36 ± 0.11	0.37 ± 0.16	0.29	0.77
EO	0.12 ± 0.08	0.11 ± 0.11	0.59	0.55
BA	0.029 ± 0.017	0.027 ± 0.016	1.86	0.07
PLT	225.76 ± 66.34	248.96 ± 52.80	1.97	0.05
ALT	21.84 ± 11.56	24.35 ± 14.62	0.97	0.34
AST	19.74 ± 14.04	23.40 ± 16.08	1.24	0.22
TP	72.08 ± 4.71	72.71 ± 4.11	0.72	0.47
ALB	44.66 ± 3.14	45.54 ± 2.71	1.45	0.15
TBIL	12.45 ± 6.66	14.14 ± 6.19	1.33	0.19
DBIL	3.46 ± 1.81	3.85 ± 1.77	1.09	0.28
Urea	4.97 ± 1.17	5.32 ± 1.35	1.44	0.15
Cr	62.79 ± 12.62	62.53 ± 11.28	0.91	0.11
UA	331.51 ± 85.73	318.20 ± 80.33	0.80	0.43
GBL	26.87 ± 4.81	27.17 ± 2.83	0.38	0.71
A/G	1.66 ± 0.23	1.70 ± 0.19	0.95	0.34
IBIL	9.01 ± 5.01	10.26 ± 3.72	1.30	0.20
MO	5.81 ± 1.53	5.19 ± 1.25	2.25	0.03
GR	63.33 ± 9.30	62.52 ± 9.37	0.44	0.66
HGB	146.02 ± 18.09	147.73 ± 15.93	0.51	0.61
MCV	89.94 ± 9.02	89.98 ± 4.91	0.03	0.97
MCH	32.35 ± 8.50	30.76 ± 2.20	1.31	0.19
MCHC	337.39 ± 45.27	341.54 ± 10.42	0.64	0.52
RDW	48.07 ± 44.66	40.70 ± 2.13	1.19	0.24
RDW‐CV	13.71 ± 4.14	12.89 ± 0.94	1.40	0.16
PDW	20.22 ± 29.40	16.09 ± 0.38	1.01	0.31
MPV	9.77 ± 1.38	9.75 ± 1.23	0.09	0.93
P‐LCR	23.73 ± 7.71	24.71 ± 8.61	0.61	0.55
PCT	0.63 ± 2.90	0.24 ± 0.04	0.96	0.34
HCT	0.42 ± 0.06	0.43 ± 0.04	1.46	0.15

*Note:* The unit of WBC, NEUT, LY, MONO, EO, BA and PLT is ×10^9^/L. The unit of ALT and AST is U/L. The unit of TP, ALB, GBL, HGB and MCHC is g/L. The unit of TBIL, DBIL, IBIL, Cr and UA is μmol/L. The unit of MO, GR, RDW‐CV, P‐LCR and PCT is %. The unit of MCV, RDW, PDW and MPV is fL. The units of MCH and HCT are pg. and L/L, respectively. The units of RBC and Urea are ×10^12^/L and mmol/L, respectively. **The details of antiepileptic therapy were not explained ensured the table was clear**. On the one hand, IRE patients received standardized antiepileptic therapy before surgery (i.e., levetiracetam tablets, 0.5 g twice daily) and continued taking levetiracetam for one year postoperatively, after which the dosage was gradually tapered over two weeks. If seizures recurred, patients resumed levetiracetam (0.5 g twice daily) and attempted medication withdrawal again after one year. IRnE patients received the same drug postoperatively (0.5 g twice daily) for three months, followed by gradual tapering over two weeks. If seizures occurred, these patients also resumed levetiracetam and attempted another withdrawal after one year. On the other hand, the review of medical records confirmed that patients did not receive anti‐inflammatory drugs (prednisone/aspirin) or antibiotics (cephalosporins) in long term before surgery. The above details may affect peripheral blood components.

Abbreviations: A/G, albumin/globulin ratio; ALB, albumin; ALT, alanine aminotransferase; AST, aspartate aminotransferase; BA, basophil; Cr, creatinine; DBIL, direct bilirubin; EO, eosinophil; GBL, globulin; GR, the percentage granulocytes; HC, healthy control; HCT, haematocrit; HGB, hemoglobin; IBIL, indirect bilirubin; IRE, insular glioma related epilepsy; IRnE, insular glioma without epilepsy; LY, lymphocyte; MCH, mean corpuscular hemoglobin; MCHC, mean corpuscular hemoglobin concentration; MCV, mean corpuscular volume; MO, the percentage of monocytes; MONO, monocyte; MPV, mean platelet volume; NEUT, neutrophil; *p*, *p* value; PCT, plateletcrit; PDW, platelet distribution width; P‐LCR, platelet large cell ratio; PLT, platelet; RBC, red blood cell; RDW, red cell distribution width; RDW‐CV, red cell distribution width‐coefficient of variation; SD, Standard Deviation; TBIL, total bilirubin; TP, total protein; UA, uric acid; Urea, blood urea nitrogen; WBC, white blood Cell.

### 3.2 Morphometry Exploration

#### 3.2.1 VBM And SBM Analysis

**FIGURE 1:**
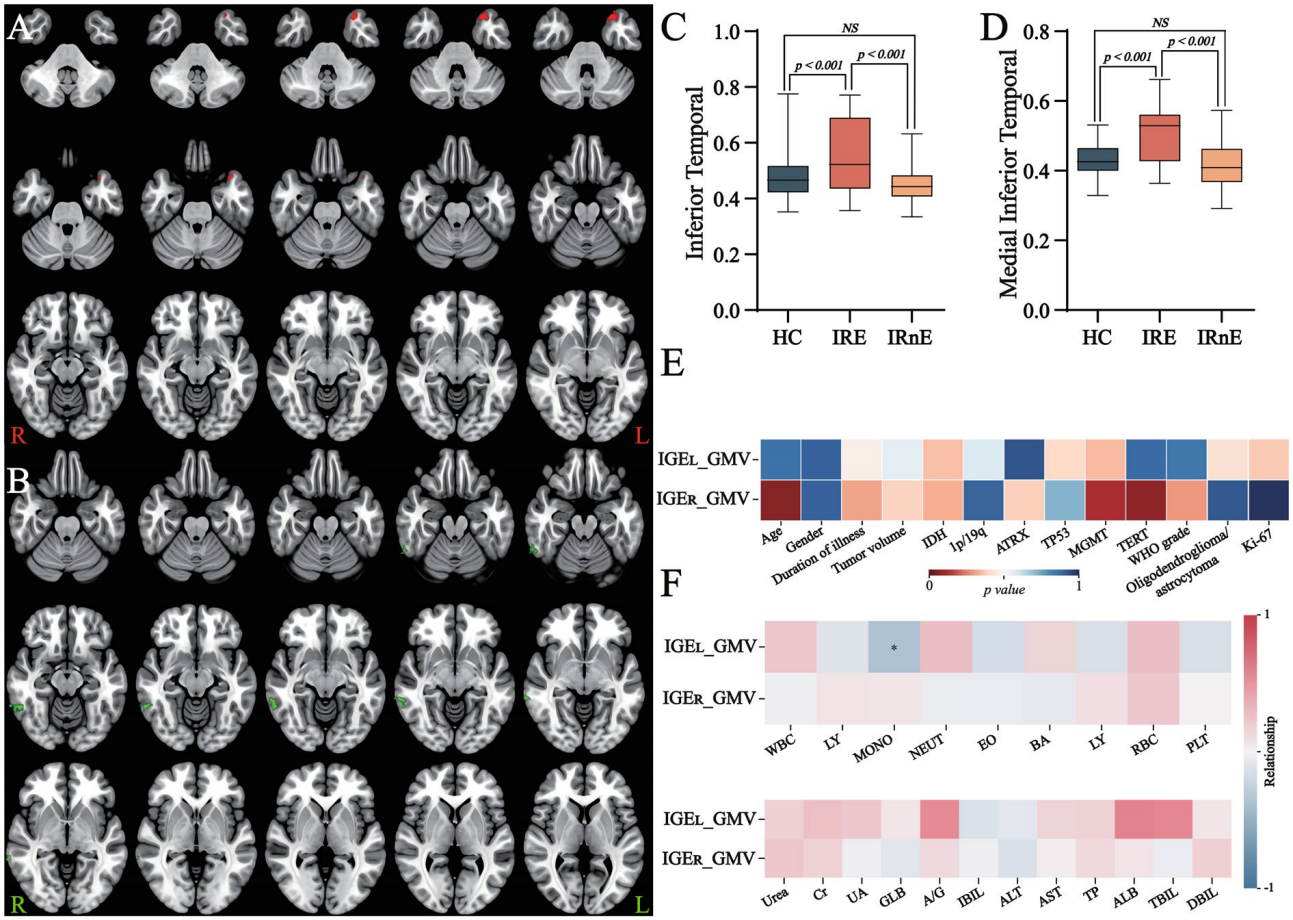
Voxel‐based morphometry analysis. (A) Potentially reorganized increased gray matter volume regions in IRE patients (tumors located on the left, IRE_L); (B) Potentially reorganized gray matter regions in IRE patients (tumors located on the right, IRE_R). (C) Post hoc testing (IRE_L). (D) Post hoc testing (IRE_R). (E) Relationship between recombination regions and clinical information. (F) Relationship between recombination regions and peripheral blood components. IRE: Insular glioma related epilepsy.

**FIGURE 2:**
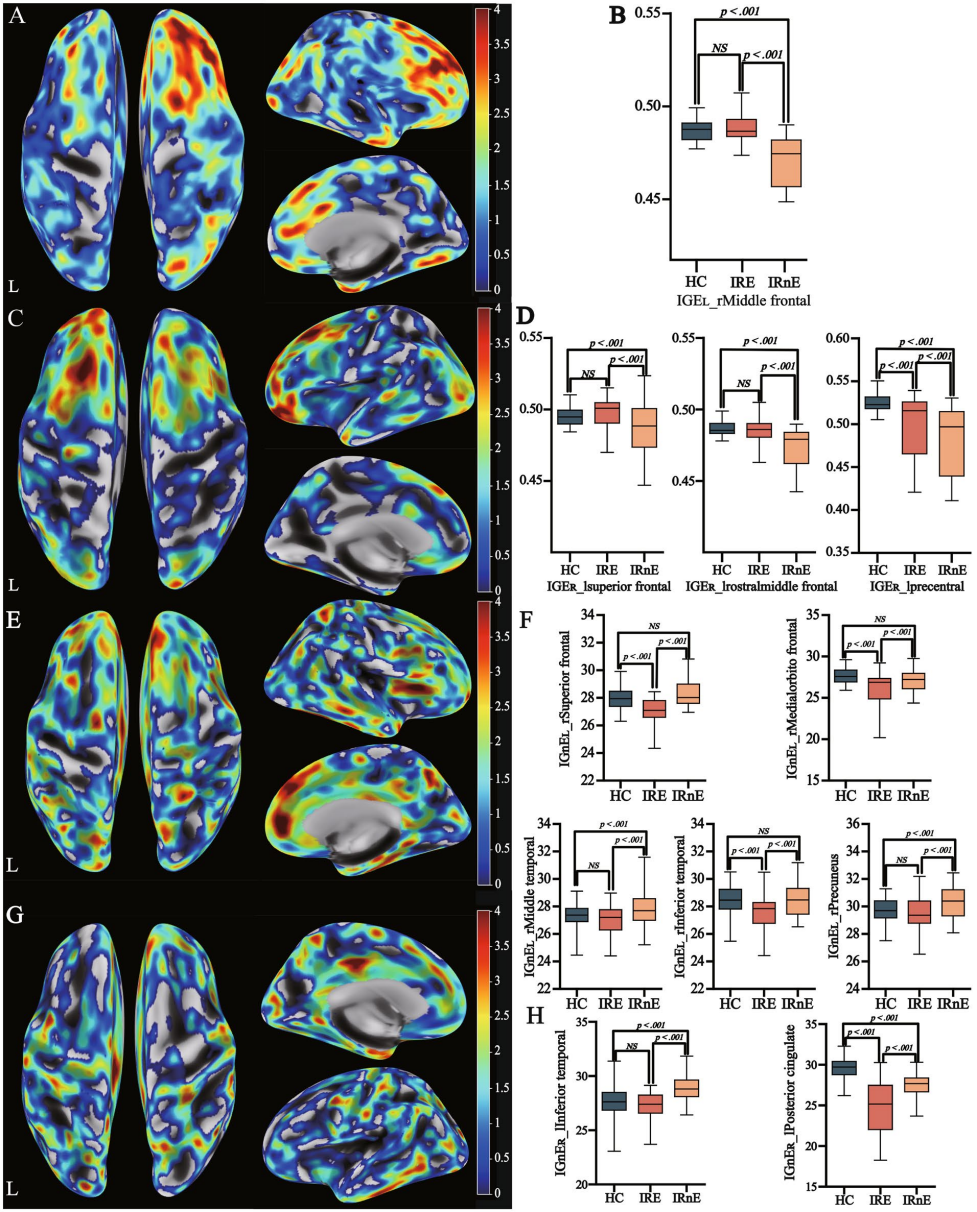
Surface‐based morphometry analysis. (A) Potential reorganization explored by increased *Toro GI* in IRE_L patients. (B) Post hoc testing (IRE_L). (C) Potential brain reorganization explored by increased *Toro GI* in IRE_R patients. (D) Post hoc testing (IRE_R). (E) Potential brain reorganization explored by increased *GI* in IRnE_L patients. (F) Post hoc testing (IRnE_L) (G) Potential brain reorganization explored by increased *GI* in IRnE_L patients. (H) Post hoc testing (IRnE_R). IRE: Insular glioma related epilepsy; IRnE: Insular tumor without epilepsy; IRE_L: IRE with tumors in the left hemisphere; IRE_R: IRE with tumors in the right hemispher; IRnE_L: IRnE with tumors in the left hemisphere; IRnE_R: IRnE with tumors in the right hemisphere; *GI*: Gyrification; Toro *GI*: Toroidal *GI*.

The whole‐brain VBM analysis demonstrated that there was no significant differential region among the three groups. Considering the influence of tumor location, we conducted further subgroup analyses. Compared with the left IRnE (IRnE_L), the left IRE (IRE_L) showed increased GMV in the ipsilateral inferior temporal region (Figure 1A,C; Table S2). Compared with the right IRnE (IRnE_R), the right IRE (IRE_R) exhibited elevated GMV in the ipsilateral medial and inferior temporal regions (Figure 1B,D; Table S2). Similarly, there were no significant cortical differences among the three groups in the whole‐brain SBM analysis. Combined with the tumor located side, subgroup analyses revealed increased *Toro GI* in the contralateral hemisphere of the IRE group, particularly the right inferior frontal cortex in IRE_L and left superior frontal, middle frontal, and precentral cortices in IRE_R (Figure 2A–D). Meanwhile, elevated *GI* was observed in the contralateral side of IRnE, including the right superior frontal, middle frontal, middle temporal, inferior temporal, and precuneus cortices in IRnE_L, as well as the left inferior temporal and posterior cingulate regions in IRnE_R (Figure 2E–H). It meant that IRE and IRnE exhibited different reorganization models. Detailed coordinates are summarized in Table S2.

Subsequently, we explored the potential impact of tumor characteristics on brain recombination and conducted further regression analyses. Univariate analyses revealed that *Toro GI* tended to be higher when tumor features included MGMT^+^, TP53^−^, IDH^−^, ATRX^**−**^, a higher WHO grade, and an elevated Ki‐67 level (Figure S4). Increased *GI* was also associated with TP53^+^, IDH^−^, a higher WHO grade, and an elevated Ki‐67 level (Figure S5). However, multivariate analyses indicated that these tumor characteristics did not influence brain reorganization. Subsequently, we regarded IRE and IRnE as different wholes and performed additional analyses. The results would not be reversed still (Tables [Link], [Link]). In addition, the increased GMV was not associated with tumor features (Figure 1E).

### 3.3 Regression Analysis

To explore the potation for postoperative events, we regarded the reorganization regions as two wholes based on the tumor side again and conducted follow‐up analyses (Table S19). Regression analyses demonstrated that increased *Toro GI* was associated with a relatively lower risk of postoperative epilepsy (IRE_L: Correlation_seizure_ = −0.14; IRE_R: Correlation_seizure_ = −0.09; both *p* < 0.05). The predictive performances of postoperative epilepsy were 0.75 (IRE_L) and 0.67 (IRE_R). Although the increased *Toro GI* could also predict tumor recurrence (IRE_L: Correlation_relapse_ = −0.27; IRE_R: Correlation_relapse_ = −0.35; both *p* < 0.05), the predictive accuracy appeared overfitted (prediction value: 1.0) due to the limited sample size (Figure S6). Similar prediction values were not discovered in GMV or *GI* in IRnE (Table S20).

### 3.4 Correlation Analysis

**FIGURE 3:**
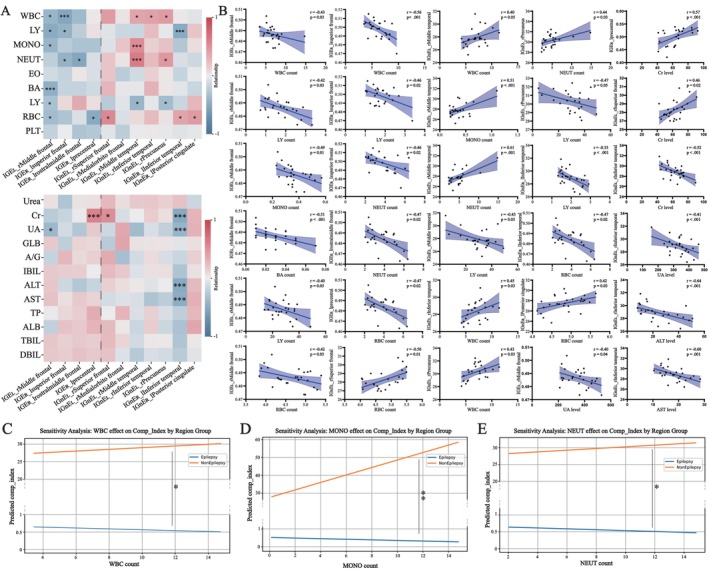
Relationship between brain reorganization described by *Toro GI* or *GI* and peripheral blood components. (A) Heatmap. (B) Detailed *t* test of the statistically significant difference. (C–E) Detailed regression analyses of the statistically significant difference (i.e., WBC, MONO, and NEUT) after PCA dimensionality reduction. PCA: Principal component analysis; *: *p* < 0.05; **: *p* < 0.01; ***: *p* < 0.001.

We explored the associations between peripheral blood components and brain reorganization and identified various relations in different recombination regions. As for IRE_L, increased *Toro GI* in the right inferior frontal cortex was negatively correlated with WBC counts. As for IRE_R, a similar association was observed between WBC counts and increased *Toro GI* in the left superior frontal and middle frontal cortices. In contrast, for IRnE_L, increased *GI* in the superior frontal, middle temporal, inferior temporal, and precuneus cortices was positively correlated with WBC counts. Additionally, several peripheral blood components, particularly monocytes (MONO) and neutrophils (NEUT), were also associated with some reorganization regions (Figure 3A,B; Figure 1F; Table S21). Subsequently, we regarded the regions in IRE and IRnE as two wholes again and explored the potential associations in the entire aspect (Table S19). For IRE, increased *Toro GI* was negatively associated with WBC, MONO, and NEUT counts. Conversely, the relationships between increased *GI* and the counts of WBC, MONO, and NEUT were opposite in IRnE (Figure 3C–E; Table S22). Although other indicators, such as lymphocyte, creatinine, alanine aminotransferase, and aspartate aminotransferase, may be related to the reorganization process in specific regions, there was no significant association between them and brain reorganization at the whole level (Table S23). Finally, we conducted an initial exploratory analysis and tried to analyze the directional relationship between brain reorganization and specific peripheral inflammatory cells. Analyses supposed that the causal direction was more likely interpreted as inflammatory cells influencing brain reorganization (indicated by higher AIC, BIC, and R^2^ values; Table S24). However, due to the lack of longitudinal studies, these results should be interpreted cautiously. In other words, the influence of brain reorganization on inflammatory cells may also objectively exist (Table S24).

## 4 Discussion

Previous studies supported that the brain possessed sufficient plasticity to adapt to different epileptic subtypes [22]. Although insular glioma‐related epilepsy (IRE) occurred frequently, morphological analyses of IRE were fewer due to the tumor mass effect. Benefited from virtual brain grafting and deep learning, we eliminated the occupancy effect, reconstructed the tumor‐involved regions, and explored potential structural changes [12, 13]. Results indicated that the brain could conduct distinct reorganization models when facing IRE and IRnE. Regression analyses demonstrated that the effects of tumor characteristics were relatively limited. Remarkably, the recombination process may be associated with the peripheral counts of white blood cells (WBC), neutrophils (NEUT), and monocytes (MONO). In other words, preoperative peripheral blood components (i.e., specific inflammatory cells) may serve as noninvasive and promising predictors of brain reorganization and could provide new insights to understand IRE and offer suggestions in the future therapy process.

From morphological analyses, we observed a relatively decreased *GI* in IRE. Previous studies revealed similar cortical alterations in epilepsy [23, 24, 25, 26]. It meant that epilepsy could affect the brain structure in multiple aspects. More importantly, we identified increased GMV and elevated *Toro GI* in several IRE regions, located in the ipsilateral gray matter and contralateral cortices, respectively. As a more precise measure, *Toro GI* may be more sensitive to subtle and spatially restricted changes in cortical folding complexity and may reflect localized compensatory remodeling or neuroplastic responses triggered by seizure [27, 28]. Additionally, considering about increased *GI* in IRnE, glioma and the mass effect may induce distinct reorganization models adapting structural distortions. We supported that there was a delicate balance between brain structural damage and compensatory reorganization in IRE and IRnE. Current research supported the recombination process from other perspectives [29, 30]. Although univariate regression analyses indicated that brain recombination was associated with tumor characteristics, such as IDH status and WHO grade, these associations were not observed in the multivariate analyses after controlling for variable interactions. To ensure stability of the results, we regarded the positive regions of IRE and IRnE as two wholes and conducted additional analyses. The results remained consistent. Therefore, we considered that the observed recombination patterns in IRE and IRnE were relatively independent of the underlying tumor features. From this perspective, clinicians may be able to classify insular gliomas into distinct subtypes based on different symptoms and understand the brain recombination process better. Further analyses indicated that increased *Toro GI* had predictive value for postoperative epilepsy in IRE. It supported that the brain could perform adaptive responses to seizures indirectly and provided valuable suggestions for future clinical management and a personal strategy for administration and withdrawal of antiepileptic drugs.

From correlation analyses, we tried to explore the associations between preoperative peripheral blood components and brain recombination and identify the potential predictive biomarkers in this non‐invasive method. Analyses indicated that *Toro GI* and *GI* were associated with the peripheral counts of WBC, NEUT, and MONO. Some studies supported our results. Zhuo et al. (2023) regarded that peripheral inflammation was associated with brain atrophy and cognitive decline [31]. Nakamichi et al. (2025) suggested that plasma cytokine was associated with hippocampal volume in Alzheimer's disease [32]. These findings supported that, as a promising noninvasive method, peripheral blood examination may reflect brain reorganization in specific disease models. When considered with the *Toro GI* value, these biomarkers or their combinations may help clinicians predict postoperative epilepsy more accurately and provide additional suggestions for the surgical and antiepileptic strategy. Additionally, as mentioned by the Society for Neuro‐oncology consensus, the withdrawal of antiepileptic drugs currently lacks uniform standards and usually relies on EEG results, which offers relatively limited clinical support [33]. In the future, with the implementation of larger prospective cohort studies, the optimal cut‐off values and the feasibility of these peripheral biomarkers could be further validated and potentially incorporated into more reliable clinical prediction models to support clinical decision‐making in different stages.

### 4.1 The Relationships Between Peripheral Blood Inflammation and Brain Reorganization in IRE and IRnE

**FIGURE 4:**
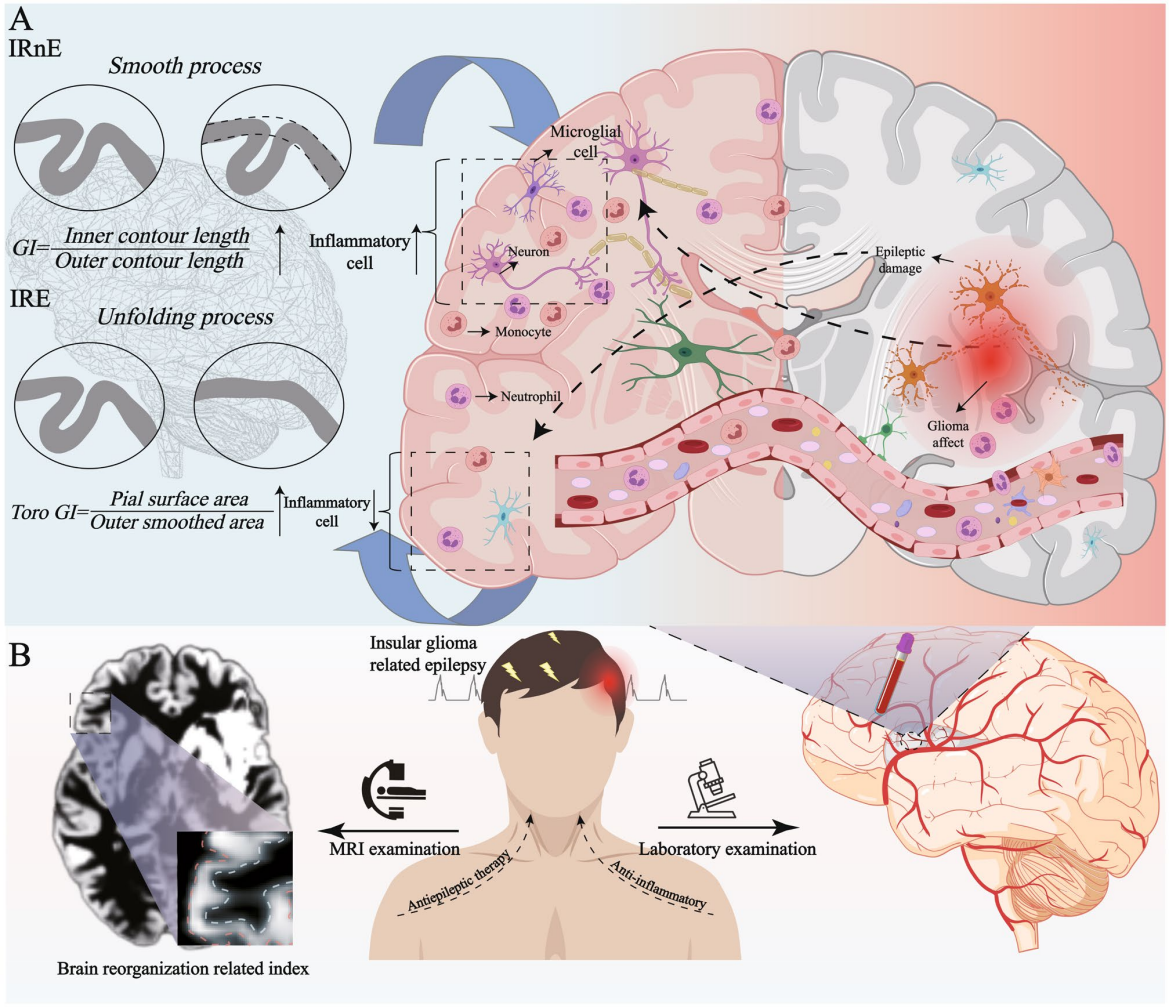
Mechanism hypothesis. (A) IRE and IRnE could conduct different brain reorganization models. In IRnE, glioma could invade adjacent normal tissues gradually and induce a relatively mild inflammatory response. The recombination (i.e., increased *GI*) may be positively associated with the counts of specific peripheral inflammatory cells. In contrast, IRE could cause overexcitation due to blood brain blood disruption, the influence of peripheral inflammation, glutamate accumulation, and brain network damage. The recombination (i.e., increased Toro *GI*) may be negatively associated with the counts of specific peripheral inflammatory cells. (B) When patients presented to the hospital with IRE, clinicians could predict potential brain reorganization and the risk of postoperative epilepsy through MRI and specific peripheral inflammatory cells. This may provide valuable suggestions for future clinical management and personalized strategies for administration and withdrawal of antiepileptic drugs. IRE: Insular glioma related epilepsy; IRnE: Insular tumor without epilepsy; *GI*: Gyrification; Toro *GI*: Toroidal *GI*.

Unexpectedly, it seemed that the associations between reorganization and peripheral inflammation components (i.e., WBC, NEUT, and MONO) appeared to differ between IRE and IRnE (Figure 4). Several studies reported the complex relationships. For instance, Núñez et al. (2019) suggested that the increased neutrophil count was associated with brain tissue loss in psychosis [34]. Gu et al. (2017) regarded that the elevated circulating C‐reactive protein level was related to cortical thickness [35]. Tortorella et al. (2014) supported a balance between pro−/anti‐ inflammatory activities of peripheral MONO and gray matter volume in multiple sclerosis [36]. These findings indicated that the effects of inflammation may vary across different disease patterns and stages.

From a biological aspect, epilepsy may disrupt the blood brain barrier (BBB) within a relatively short period of time in IRE [37]. Peripheral inflammatory cells and related factors (e.g., monocyte and IL‐1β) could affect the cellular components of the BBB or directly cross it, activate microglia, and increase excessive neuroinflammation [38, 39]. Previous studies supported that the overactive responses could inhibit neuronal dendritic growth, myelinization, and synaptic remodeling [40, 41, 42]. Meanwhile, some inflammatory cytokines could directly damage neural plasticity pathways through complex molecular interactions [38]. Additionally, epilepsy may cause the overactivation of glutamate receptors, resulting in neuronal excitotoxicity [43, 44]. Current research demonstrated that GLT‐1, a crucial transporter protein, could remove glutamate from the synaptic cleft and prevent its excessive accumulation. Some studies revealed that elevated peripheral inflammatory cells would impair the function of GLT‐1 mediated by astrocytes, leading to further neuronal excitotoxicity [43, 45, 46]. From a topological aspect, imaging studies showed that seizures may affect the brain network level and induce structural reorganization in other regions. Aruldass et al. (2021) reported that peripheral NEUT counts were negatively associated with functional connectivity in depression [47]. Thus, peripheral inflammation may reduce the connection strength between the epileptic focus and potential reorganized areas. We speculated that these overexcited responses may explain why peripheral inflammatory cell counts were negatively associated with brain organization in IRE. Unlike IRE, IRnE patients underwent medical examinations during routine health check‐ups or when experiencing chronic headaches or gradually declining limb muscle strength. Glioma may invade adjacent normal tissues gradually and induce chronic systemic inflammatory responses in a relatively stable state. Current studies supported that chronic peripheral inflammation could promote revascularization, oligodendrocyte precursor cell (OPC) proliferation, and enhance myelin plasticity, thereby facilitating brain plasticity and reorganization [44, 48]. For instance, Poletti et al. (2019) found that peripheral cytokine levels were positively associated with cortical thickness in bipolar patients affected by chronic inflammation [49]. Lizano et al. (2021) reported similar associations in psychosis [50]. Besteher et al. (2024) revealed that long‐COVID could create a proinflammatory environment in peripheral blood and prime intracranial microglia to become hyperreactive, inducing cortical reorganization [51]. This evidence suggested that peripheral inflammation may serve as an appropriate stimulus capable of inducing brain reorganization in IRnE. Combined with the distinct immune landscapes and reorganization models, IRE and IRnE may represent distinct subtypes of insular glioma. Recognizing insular glioma from a unique perspective may offer new insights and valuable suggestions for future hierarchical disease management.

Although the causal direction was more strongly interpreted as inflammatory cells affecting brain reorganization, the objective reverse potential associations should also be emphasized. For example, brain network reorganization induced by injury or stimulation may alter peripheral inflammation through several pathways or axes, such as the parasympathetic and sympathetic nervous systems, the classical HPA axis, and the efferent vagus nerve‐cholinergic signaling pathway [52, 53, 54, 55]. Clinical evidence also demonstrated the association. Xie et al. (2024) applied transcranial magnetic stimulation to 54 patients with Parkinson's disease and demonstrated that the function of the M1 motor cortex could be improved and increase peripheral regulatory T cells [56]. However, the evidence about structural reorganization (e.g., gray matter volume) remained limited. Therefore, it was necessary for future studies continue to clarify and accurately characterize the interactions within neuro‐immune regulatory pathways.

There were some limitations in our studies. Firstly, as a small population cross‐sectional study, the causal relationship between brain reorganizations and peripheral inflammatory cells was limited and should be interpreted cautiously. In the future, we should conduct large‐scale longitudinal and prospective studies deliberately among some patients who refused surgery and chose temporary observation and further identify their associations and the best cut‐off values. Secondly, some patients were unable to objectively describe their detailed symptoms and seizure frequency. Therefore, the detailed characteristics of epilepsy were not discussed in our study. The stability of our study may be potentially influenced. Thirdly, although IRE and IRnE patients received suitable standardized antiepileptic treatment during both the preoperative and postoperative periods, the true therapeutic strategy may be potentially different due to medical and individual patient conditions. It might affect peripheral blood components and brain reorganization. Fourthly, as an exploration study, we identified several indicators and tried to draw sufficient attention to peripheral blood inflammation, especially WBC, NEUT, and MONO. However, it was necessary to identify more specific biomarkers and obtain more direct evidence, such as interleukin levels in peripheral blood or CSF. Finally, we regarded brain reorganization as two wholes in IRE and IRnE. The potential functions of individual indices in specific regions were not further explored. In fact, some blood components could also influence seizures, like serum creatinine [57].

### 4.2 Conclusion

Our study indicated that the brain exhibited different reorganization patterns in IRE (i.e., increased GMV and *Toro GI*) and IRnE (i.e., increased *GI*). *Toro GI* showed a predictive value for postoperative seizure control in IRE. Importantly, we identified associations between brain reorganization and peripheral inflammatory cells, such as WBC, NEUT, and MONO. The direction of the associations appeared to differ between IRE and IRnE. In the future, longitudinal studies are needed to further elucidate the causal relationship of neuro‐immune interactions and to identify potential specific biomarkers providing suggestions for postoperative epilepsy prediction, therapeutic strategies, and drug withdrawal criteria.

## Author Contributions

H.Z.: investigation, methodology, writing – original draft. B.Z.: investigation, methodology, writing – original draft. Q.H.: writing – review and editing. Z.D.: writing – review and editing; Z.H.: writing – review and editing; J.X.: conceptualization, funding acquisition, investigation, writing – review and editing.

## Funding

This work was supported by The National Natural Science Foundation of China, 82172028.

## Disclosure

Permission to Reproduce Material From Other Sources: Not available.

Clinical Trial Registration: Not available.

## Consent

The study was approved by the institutional review board of Beijing Tiantan Hospital, and written informed consent was obtained from all participants (KY 2020–146‐02).

## Conflicts of Interest

The authors declare no conflicts of interest.

## Supporting information


**Figure S1:** Detailed learning processes of deep learning. (A) First epoch. (B) Second epoch. (C) Third epoch. (D) Fourth epoch. (E) Fifth epoch.

**Figure S2:** Quality check process. (A) To identify that there was no serious tumor mass effect, severe or widespread brain edema and anatomical distortion in the primary data ensured. (B) To ensure that reconstructed structures could fill the tumor mask accurately. (C) To check the structure segment outcomes by quality reports. (D) To observe the potential abnormalities such as voids in the surface mesh, extreme values in the cortical thickness map, surface fractures or discontinuities, or abnormal sulcal patterns.

**Figure S3:** Detailed information of the included patients and glioma lesions. (A) Inclusion and exclusion processes for patients and healthy volunteers. (B) Overlapping region for gliomas.

**Figure S4:** Relationship between clinical information and increased *Toro GI* in IRE. (A) Heatmap. (B) Detailed *t* test of the statistically significant difference. (C) Detailed *t* test of the statistically significant difference. IRE: insular glioma related epilepsy; PCA: Principal component analysis; *GI*: gyrification; Toro GI: toroidal GI; *: *p* < 0.05; **: *p* < 0.01; ***: *p* < 0.001.

**Figure S5:** Relationship between clinical information and increased *GI* in IRnE. (A) Heatmap. (B) Detailed *t* test of the statistically significant difference. The test about MGMT and TERT is not conducted in 5 regions due to a small sample (*n =* 2). IRnE: insular tumor without epilepsy; *GI*: gyrification; *: *p* < 0.05; **: *p* < 0.01; ***: *p* < 0.001.

**Figure S6:** Regression analysis. (A) Relationship between seizure control and brain reorganization in IRE_L. (B) Relationship between tumor recurrence and brain reorganization in IRE_L. (C) Relationship between seizure control and brain reorganization in IRE_R. (D) Relationship between tumor recurrence and brain reorganization in IRE_R. IRE: insular glioma‐related epilepsy; IRE_L: IRE with tumors in the left hemisphere; IRE_R: IRE with tumors in the right hemisphere; *: *p* < 0.05; **: *p* < 0.01; ***: *p* < 0.001.

**Table S1:** MR imaging parameters.

**Table S2:** Summary of brain reorganization regions between the IRE and IRnE.

**Table S3:** Multivariable regression analysis of brain reorganization in the inferior temporal gray matter volume of IRE_L and clinical variables.

**Table S4:** Multivariable regression analysis of brain reorganization in the medial inferior temporal gray matter volume of IRE_R and clinical variables.

**Table S5:** Multivariable regression analysis of brain reorganization in the middle frontal cortex of IRE_R and clinical variables.

**Table S6:** Multivariable regression analysis of brain reorganization in the superior frontal cortex of IRE_R and clinical variables.

**Table S7:** Multivariable regression analysis of brain reorganization in the middle frontal cortex of IRE_R and clinical variables.

**Table S8:** Multivariable regression analysis of brain reorganization in the precentral cortex of IRE_R and clinical variables.

**Table S9:** Multivariable regression analysis of brain reorganization after principal component analysis of IRE_R and clinical variables.

**Table S10:** Multivariable regression analysis of brain reorganization in the superior frontal cortex of IRnE_L and clinical variables.

**Table S11:** Multivariable regression analysis of brain reorganization in the middle frontal cortex of IRnE_L and clinical variables.

**Table S12:** Multivariable regression analysis of brain reorganization in the middle temporal cortex of IRnE_L and clinical variables.

**Table S13:** Multivariable regression analysis of brain reorganization in the inferior temporal cortex of IRnE_L and clinical variables.

**Table S14:** Multivariable regression analysis of brain reorganization in the precuneus cortex of IRnE_L and clinical variables.

**Table S15:** Multivariable regression analysis of brain reorganization after principal component analysis of IRE_L and clinical variables.

**Table S16:** Multivariable regression analysis of brain reorganization in the inferior temporal cortex of IRnE_R and clinical variables.

**Table S17:** Multivariable regression analysis of brain reorganization in the posterior cingulate cortex of IRnE_R and clinical variables.

**Table S18:** Multivariable regression analysis of brain reorganization after principal component analysis of IRE_R and clinical variables.

**Table S19:** PCA derived compensation indices across different models.

**Table S20:** Correlations between the brain reorganization and postoperative events in IRE and IRnE.

**Table S21:** Correlation matrix of the brain compensation regions and potential biomarkers in peripheral blood.

**Table S22:** Regression analysis of potential biomarkers in peripheral blood.

**Table S23:** Correlation matrix of the whole brain compensation regions and potential biomarkers in peripheral blood.

**Table S24:** Analysis of potential causal link between specific peripheral blood cells and brain reorganization.

## Data Availability

The data that support the findings of this study are available from the corresponding author upon reasonable request.
