# Correction to “The Dual Associations of Peripheral Inflammatory Cells With Brain Reorganization in Insular Gliomas With/Without Epilepsy: An Exploratory Analysis”

**DOI:** 10.1002/cns.71136

**Published:** 2026-09-03

H. Zhao , B. Zhang , Q. He , Z. Deng , Z. Hou , and J. Xie , “The Dual Associations of Peripheral Inflammatory Cells With Brain Reorganization in Insular Gliomas With/Without Epilepsy: An Exploratory Analysis,” CNS Neuroscience & Therapeutics 32, no. 2 (2026): e70788, 10.1002/cns.70788.41721208 PMC12927981

The author's affiliations for Hongfang Zhao, Bohan Zhang, and Qifeng He has been incorrectly cited in the originally published version of this article. The correct citation is provided below.

Hongfang Zhao^1^ | Bohan Zhang^2^ | Qifeng He^1^ | Zhenghai Deng^1^ | Zonggang Hou^1^ | Jian Xie^1^

^1^Department of Neurosurgery, Beijing Tiantan Hospital, Capital Medical University, Beijing, China | ^2^Department of Neurology, Beijing Tiantan Hospital, Capital Medical University, Beijing, China

We apologize for this error.

