# Supplementary figures and images for: The Dual Associations of Peripheral Inflammatory Cells With Brain Reorganization in Insular Gliomas With/Without Epilepsy: An Exploratory Analysis
Source: CNS Neurosci Ther. 2026 Feb 20;32(2):e70788. doi: 10.1002/cns.70788 (PMC12927981; doi:10.1002/cns.70788)

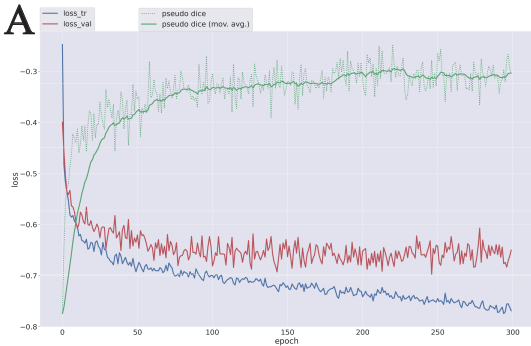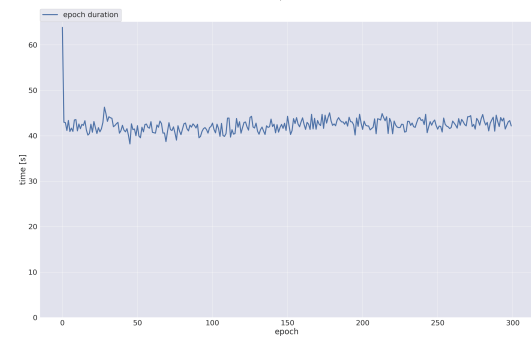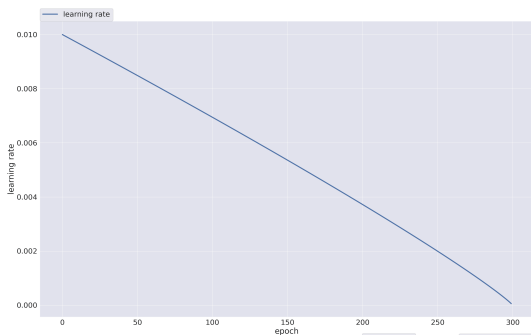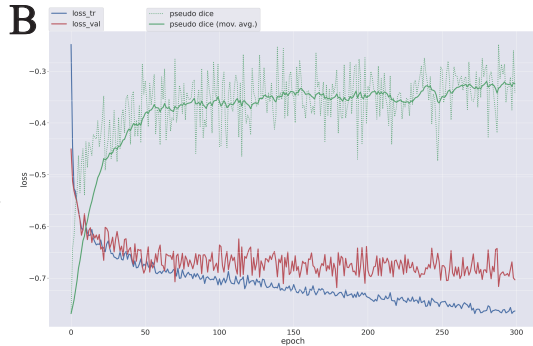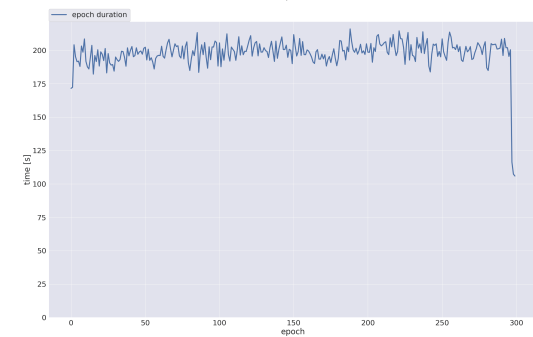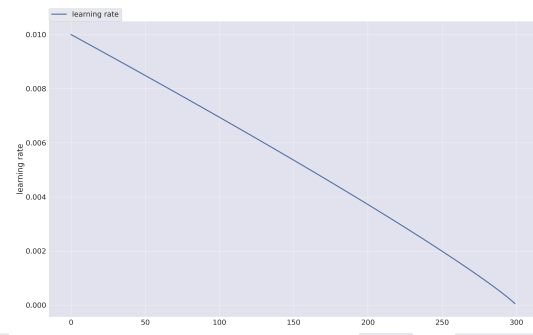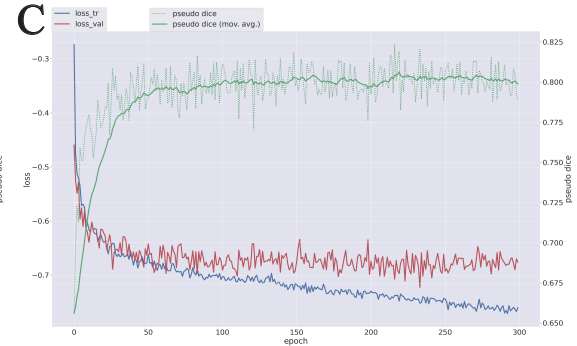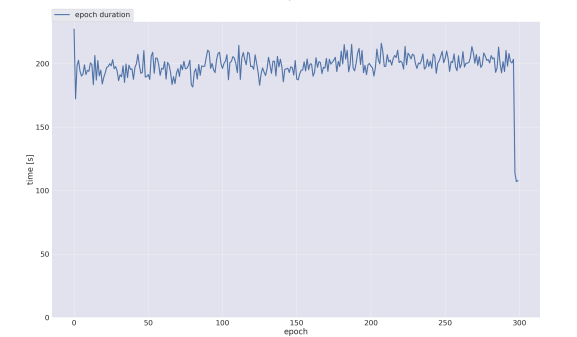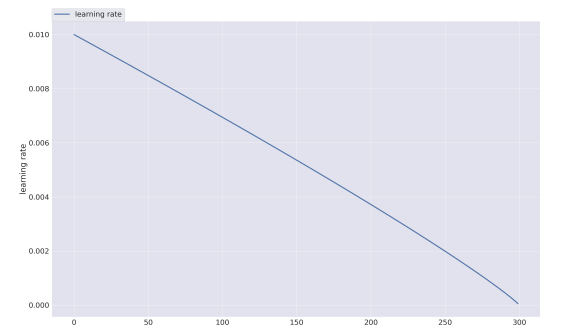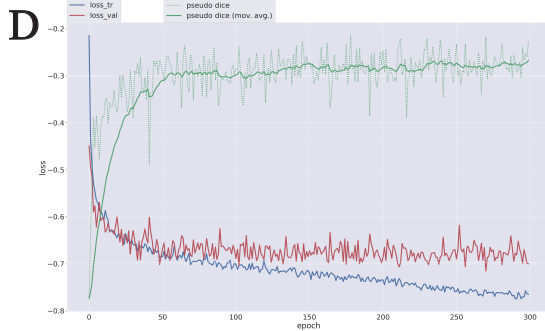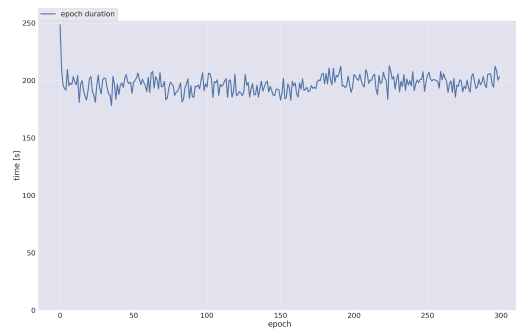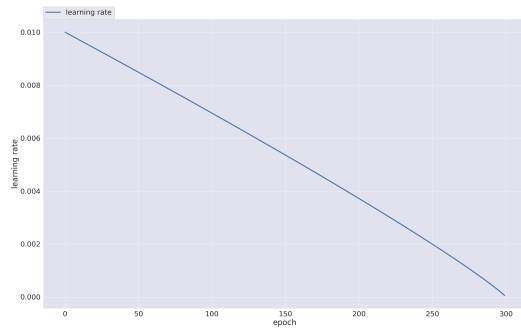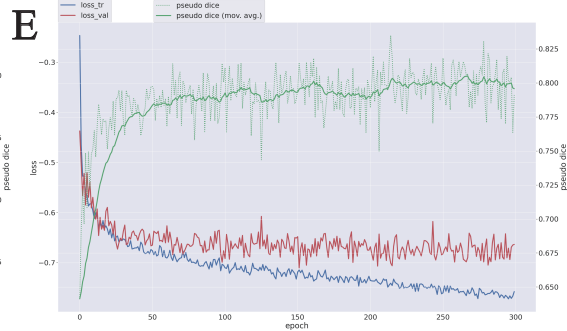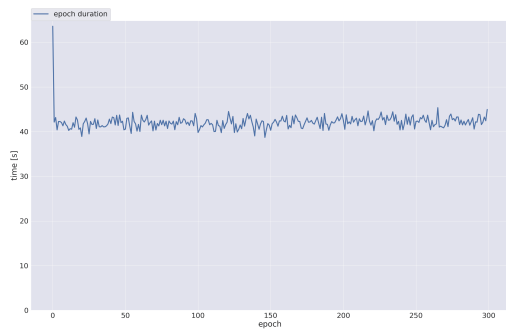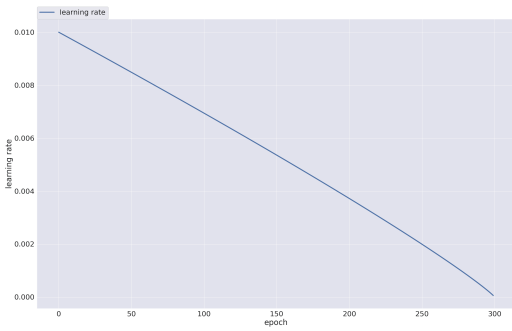

Supplement: Supplementary file 1 — Figure S1: Detailed learning processes of deep learning. (A) First epoch. (B) Second epoch. (C) Third epoch. (D) Fourth epoch. (E) Fifth epoch. [file CNS-32-e70788-s021.pdf]

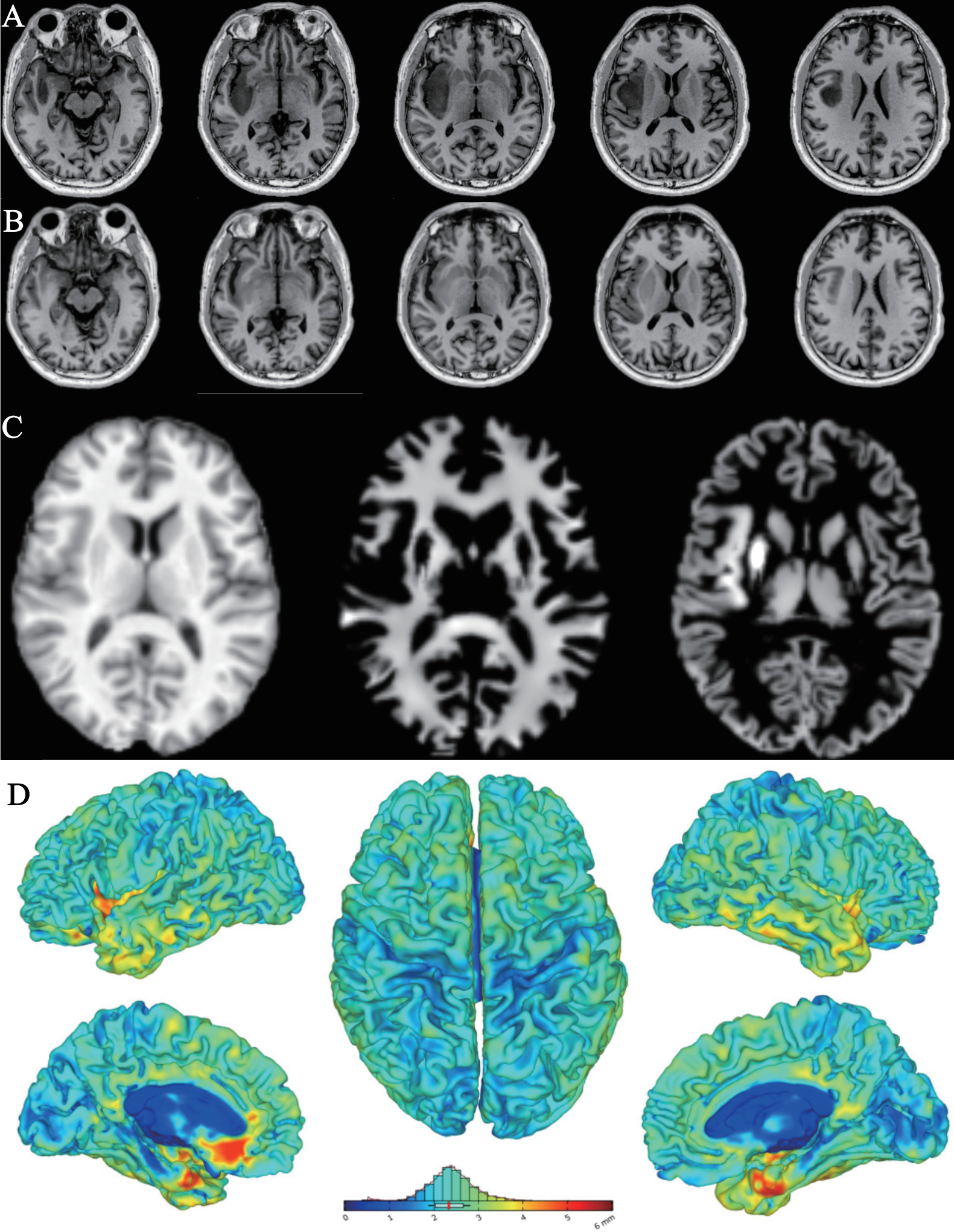

Supplement: Supplementary file 2 — Figure S2: Quality check process. (A) To identify that there was no serious tumor mass effect, severe or widespread brain edema and anatomical distortion in the primary data ensured. (B) To ensure that reconstructed structures could fill the tumor mask accurately. (C) To check the structure segment outcomes by quality reports. (D) To observe the potential abnormalities such as voids in the surface mesh, extreme values in the cortical thickness map, surface fractures or discontinuities, or abnormal sulcal patterns. [file CNS-32-e70788-s012.pdf]

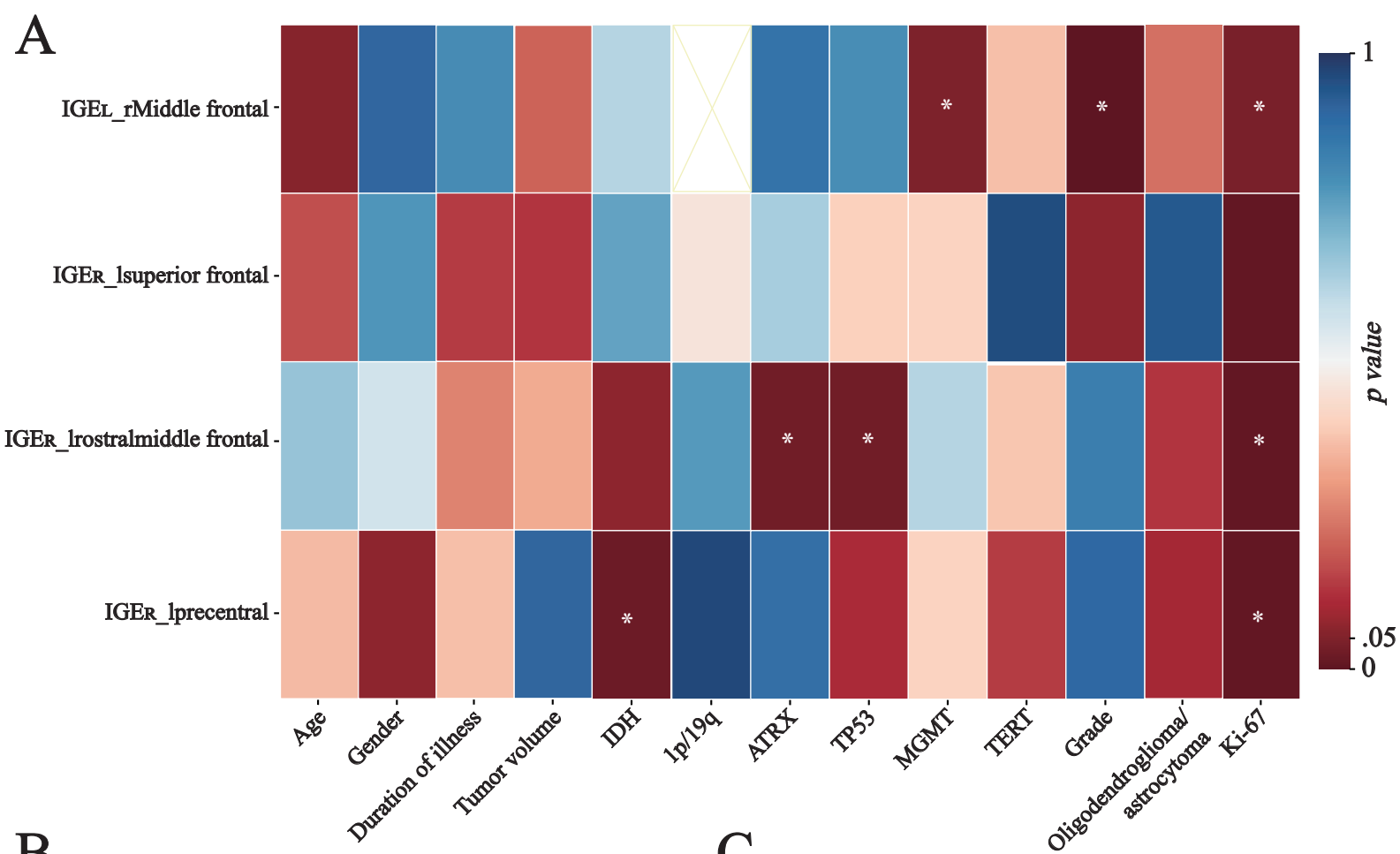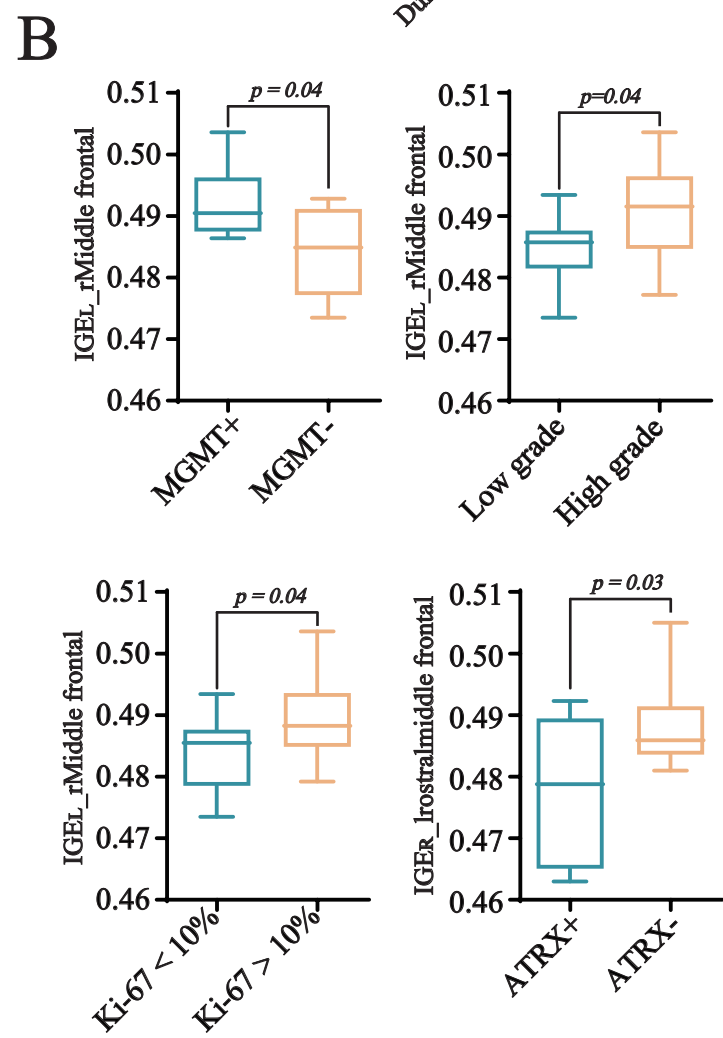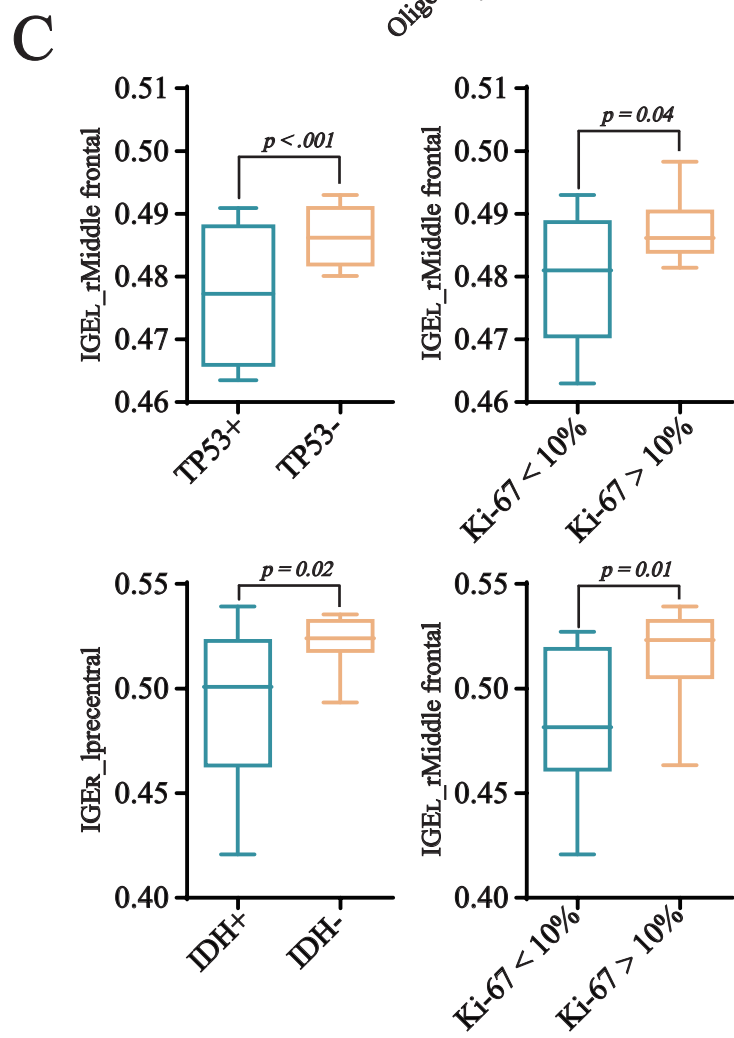

Supplement: Supplementary file 4 — Figure S4: Relationship between clinical information and increased Toro GI in IRE. (A) Heatmap. (B) Detailed t test of the statistically significant difference. (C) Detailed t test of the statistically significant difference. IRE: insular glioma related epilepsy; PCA: Principal component analysis; GI: gyrification; Toro GI: toroidal GI; *: p < 0.05; **: p < 0.01; ***: p < 0.001. [file CNS-32-e70788-s005.pdf]

A

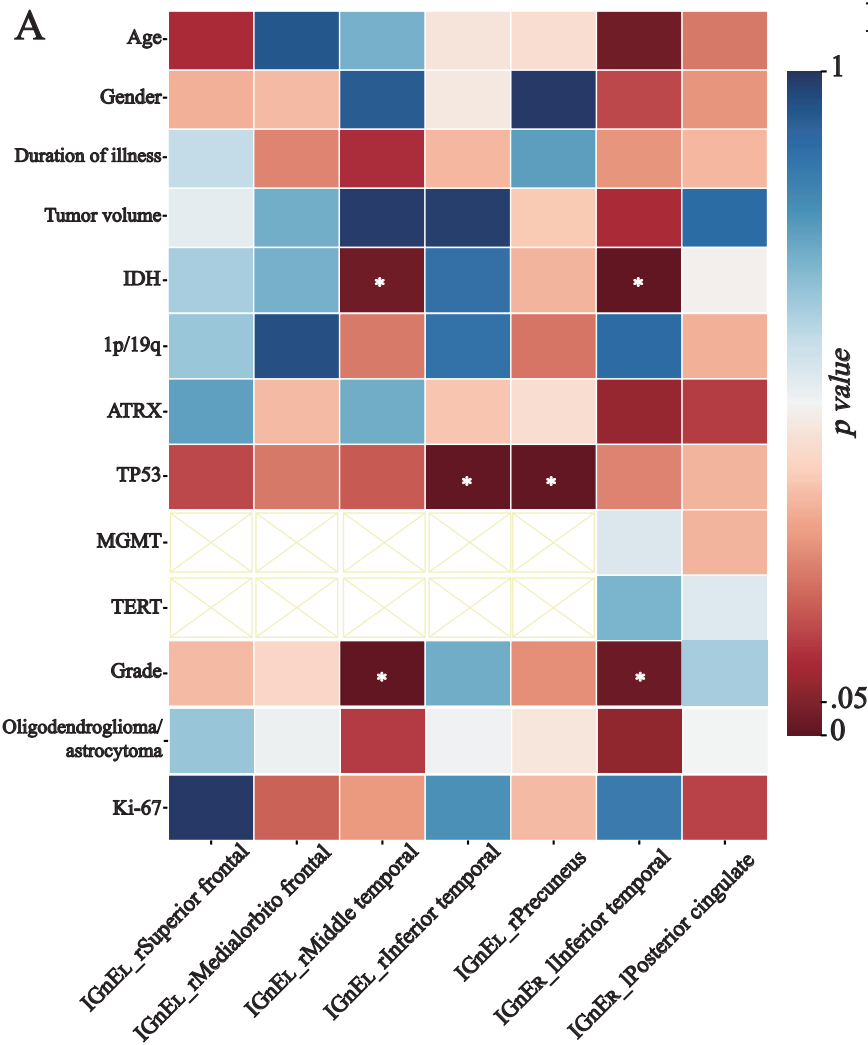

B

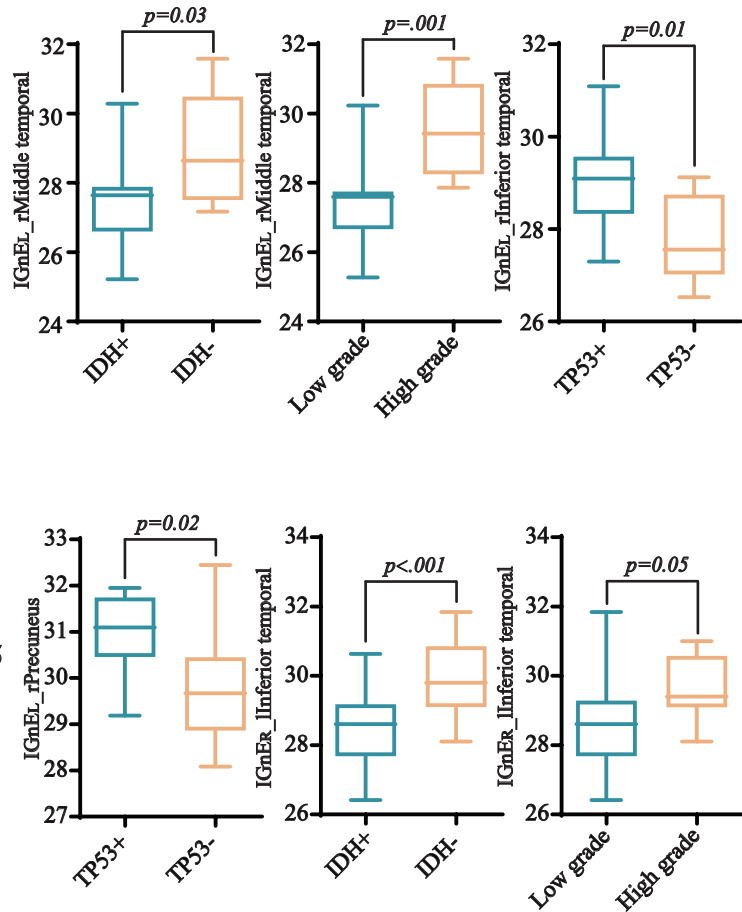

Supplement: Supplementary file 5 — Figure S5: Relationship between clinical information and increased GI in IRnE. (A) Heatmap. (B) Detailed t test of the statistically significant difference. The test about MGMT and TERT is not conducted in 5 regions due to a small sample (n = 2). IRnE: insular tumor without epilepsy; GI: gyrification; *: p < 0.05; **: p < 0.01; ***: p < 0.001. [file CNS-32-e70788-s029.pdf]

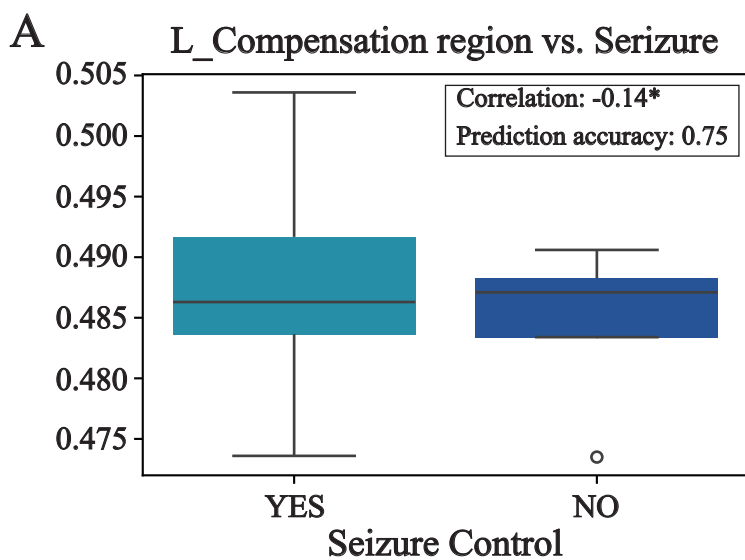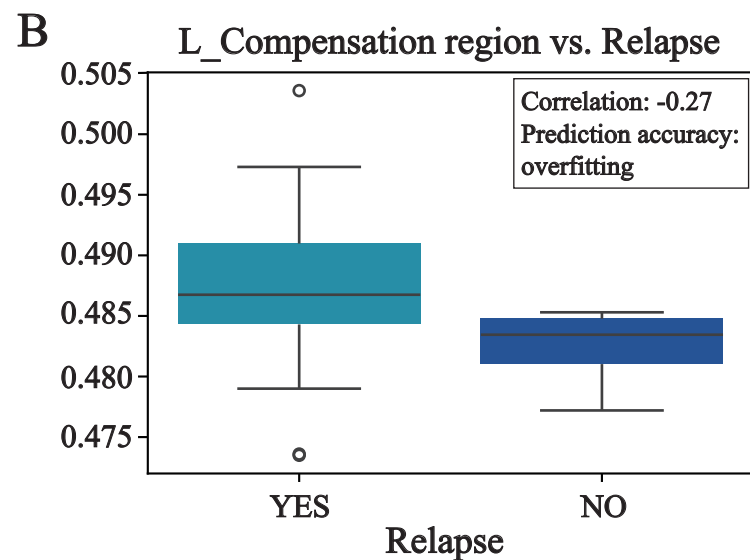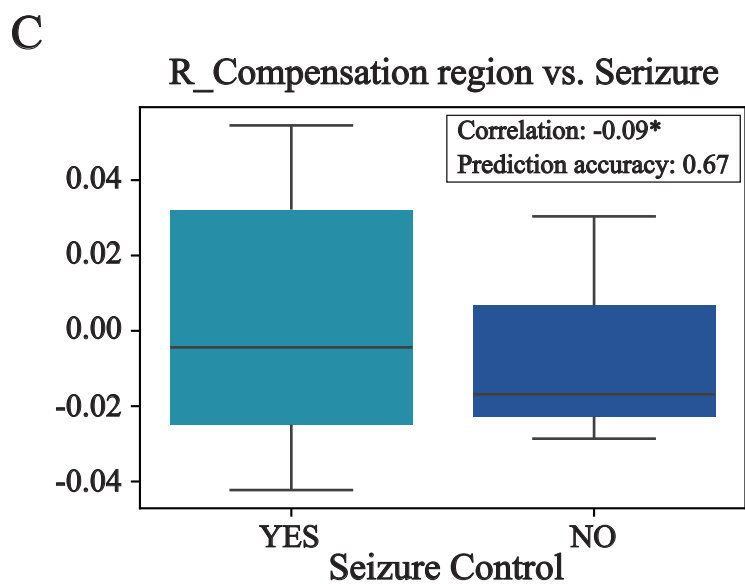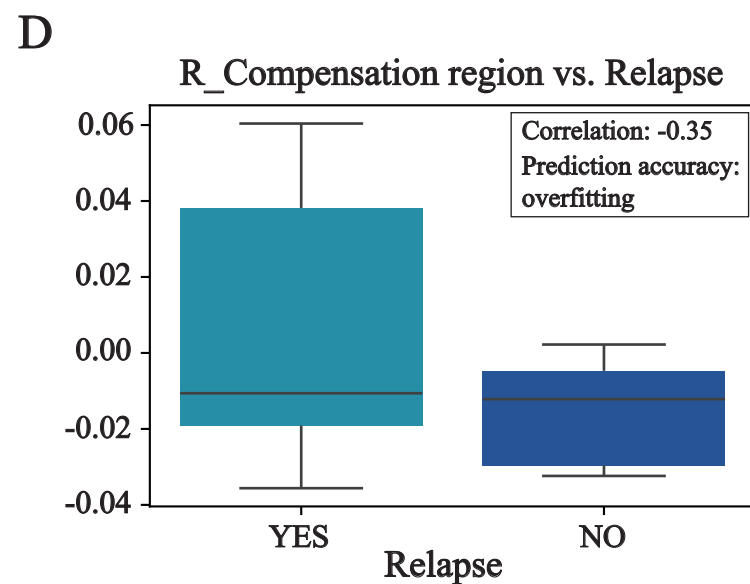

Supplement: Supplementary file 6 — Figure S6: Regression analysis. (A) Relationship between seizure control and brain reorganization in IRE_L. (B) Relationship between tumor recurrence and brain reorganization in IRE_L. (C) Relationship between seizure control and brain reorganization in IRE_R. (D) Relationship between tumor recurrence and brain reorganization in IRE_R. IRE: insular glioma‐related epilepsy; IRE_L: IRE with tumors in the left hemisphere; IRE_R: IRE with tumors in the right hemisphere; *: p < 0.05; **: p < 0.01; ***: p < 0.001. [file CNS-32-e70788-s027.pdf]
