# Supplementary material for: The Dual Associations of Peripheral Inflammatory Cells With Brain Reorganization in Insular Gliomas With/Without Epilepsy: An Exploratory Analysis
Source: CNS Neurosci Ther. 2026 Feb 20;32(2):e70788. doi: 10.1002/cns.70788 (PMC12927981; doi:10.1002/cns.70788)
Supplement: Supplementary file 3 — Figure S3: Detailed information of the included patients and glioma lesions. (A) Inclusion and exclusion processes for patients and healthy volunteers. (B) Overlapping region for gliomas. [file CNS-32-e70788-s023.pdf]

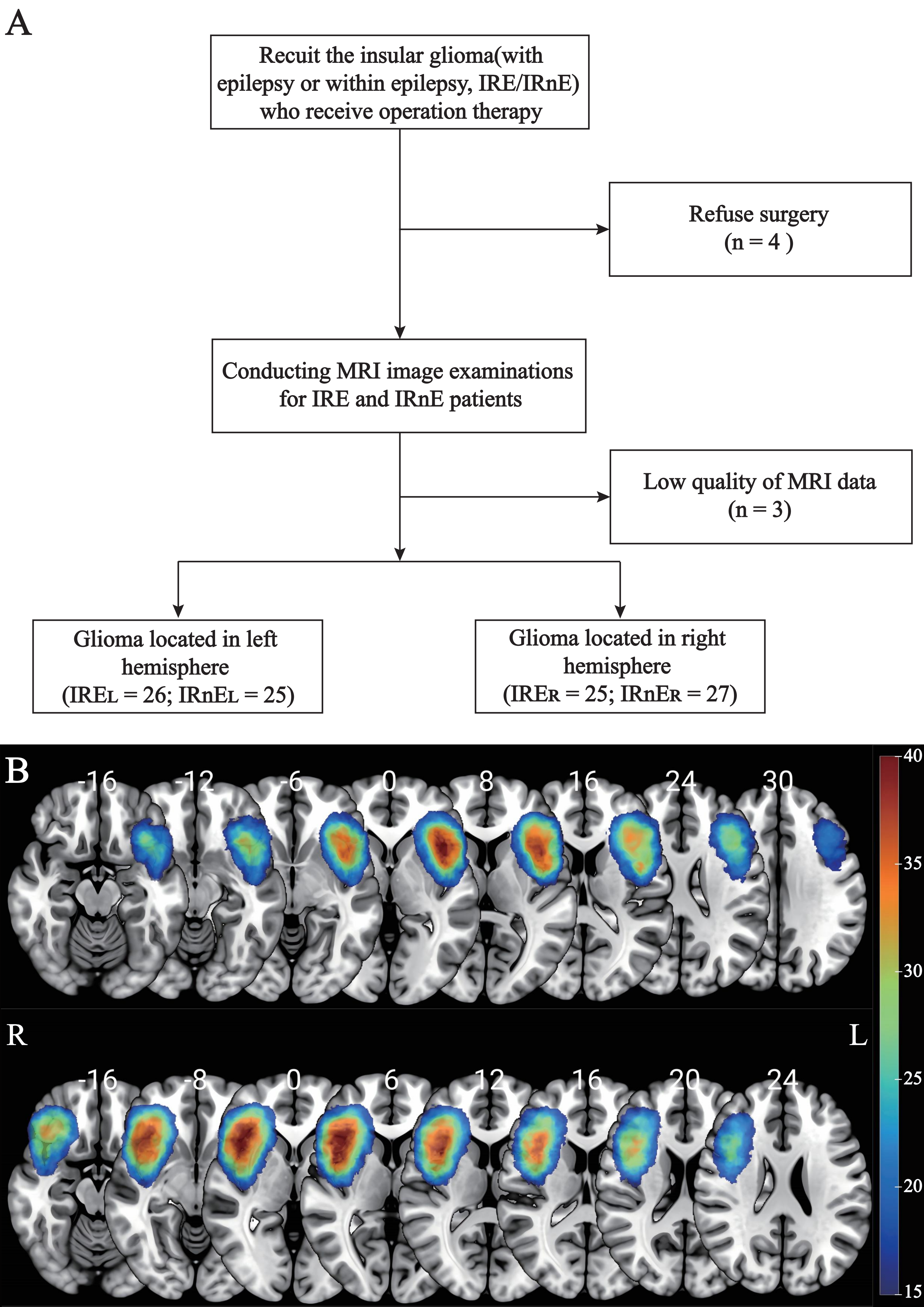
A
Recuit the insular glioma(with epilepsy or within epilepsy, IRE/IRnE) who receive operation therapy
Refuse surgery (n = 4)
Conducting MRI image examinations for IRE and IRnE patients
Low quality of MRI data (n = 3)
Glioma located in left hemisphere (IREL = 26; IRnEL = 25)
Glioma located in right hemisphere (IRER = 25; IRnER = 27)
B
-16 -12 -6 0 8 16 24 30
R
L
-16 -8 0 6 12 16 20 24
40
35
30
25
20
15
