# Supplementary material for: The Dual Associations of Peripheral Inflammatory Cells With Brain Reorganization in Insular Gliomas With/Without Epilepsy: An Exploratory Analysis
Source: CNS Neurosci Ther. 2026 Feb 20;32(2):e70788. doi: 10.1002/cns.70788 (PMC12927981; doi:10.1002/cns.70788)
Supplement: Supplementary file 7 — Table S1: MR imaging parameters. [file CNS-32-e70788-s020.docx]

**Table S1. MR Imaging Parameters.**

| **Sequence type** | **TR (ms)** | **TE (ms)** | **TI (ms)** | **Flip angle (°)** | **Matrix size** | **FOV (mm)** | **Slice thickness (mm)** | **Notes** |
| --- | --- | --- | --- | --- | --- | --- | --- | --- |
| 3D T1-weighted (MPRAGE) | 1540 | 2.4 | 900 | 8 | 256 × 232 | 217 × 240 | 1 | Pre-contrast |
| T2-weighted (TSE) | 4500 | 105 | N/A | 150 | 448 × 283 | 185 × 220 | 5 | N/A |
| FLAIR (TSE) | 6000 | 81 | 2028.3 | 90 | 320 × 196 | 192 × 220 | 5 | Fluid-attenuated sequence |
| Contrast-enhanced 3D T1-weighted | 1540 | 2.4 | N/A | 8 | 256 × 232 | N/A | 1 | Gadobenate dimeglumine (0.1 mmol/kg, 4.0 mL/s + 30 mL saline flush) |
