# Supplementary material for: The Dual Associations of Peripheral Inflammatory Cells With Brain Reorganization in Insular Gliomas With/Without Epilepsy: An Exploratory Analysis
Source: CNS Neurosci Ther. 2026 Feb 20;32(2):e70788. doi: 10.1002/cns.70788 (PMC12927981; doi:10.1002/cns.70788)
Supplement: Supplementary file 8 — Table S2: Summary of brain reorganization regions between the IRE and IRnE. [file CNS-32-e70788-s026.docx]

**Table S2. Summary of brain reorganization regions between the IRE and IRnE.**

| **Analysis method** | **Tumor side** | **Research index** | **Brain region** | **Peak MNI**  **coordinates** | | | **Cluster size**  **(voxels)** | **Peak**  **intensity** |
| --- | --- | --- | --- | --- | --- | --- | --- | --- |
|  |  |  |  | **X** | **Y** | **Z** |  |  |
| VBM^a^ | L | GMV | Inferior temporal | 62 | -50 | -9 | 273 | 4.20 |
|  | R |  | Medial Inferior temporal | -33 | 16 | -36 | 158 | 4.10 |
| SBM^b^ | L | *Toro GI* | Middle frontal | 49 | 30 | 29 | 615 | 4.81 |
|  | R |  | Superior frontal | -16 | 59 | 16 | 76 | 3.87 |
|  |  |  | Middle frontal | -26 | 53 | -13 | 321 | 5.42 |
|  |  |  | Precentral | -23 | 17 | 43 | 176 | 3.87 |
|  | L | *GI* | Superior frontal | 15 | 44 | 7 | 169 | 4.57 |
|  |  |  | Middle frontal | 33 | 21 | 11 | 205 | 4.72 |
|  |  |  | Middle temporal | 37 | -32 | -19 | 129 | 3.64 |
|  |  |  | Inferior temporal | 40 | -65 | -17 | 115 | 3.99 |
|  |  |  | Precuneus | 6 | -65 | 42 | 106 | 4.11 |
|  | R |  | Inferior temporal | -54 | -32 | -30 | 61 | 3.92 |
|  |  |  | Posterior cingulate | -5 | -24 | 42 | 119 | 4.64 |

**Abbreviations:** VBM: Voxel-based morphometry analysis; SBM: Surface-based morphometry analysis; GMV: Grey matter volume; *GI*: Gyrification; Toro *GI*: Toroidal *GI*; L: Left; R: Right. **Some details were not shown to make the table clear.** ^a^ The reorganization side of VBM are ipsilateral hemisphere. ^b^ The reorganization side of SBM are contralateral hemisphere.
