# Supplementary material for: The Dual Associations of Peripheral Inflammatory Cells With Brain Reorganization in Insular Gliomas With/Without Epilepsy: An Exploratory Analysis
Source: CNS Neurosci Ther. 2026 Feb 20;32(2):e70788. doi: 10.1002/cns.70788 (PMC12927981; doi:10.1002/cns.70788)
Supplement: Supplementary file 9 — Table S3: Multivariable regression analysis of brain reorganization in the inferior temporal gray matter volume of IRE_L and clinical variables. [file CNS-32-e70788-s018.docx]

**Table S3. Multivariable regression analysis of brain reorganization in the inferior temporal grey matter volume of IRE_L and clinical variables.**

| Variables | coef. | std. err. | t | *p* > \|t\| | 95% CI  Lower | 95% CI Upper |
| --- | --- | --- | --- | --- | --- | --- |
| Gender | 0.063 | 0.083 | 0.755 | 0.465 | -0.119 | 0.244 |
| Age | 0.002 | 0.004 | 0.458 | 0.655 | -0.007 | 0.011 |
| Time of duration | 0.013 | 0.099 | 0.132 | 0.898 | -0.203 | 0.229 |
| Tumor volume | 0.044 | 0.068 | 0.648 | 0.529 | -0.105 | 0.193 |
| *IDH* | -0.034 | 0.085 | -0.396 | 0.699 | -0.218 | 0.151 |
| *ATRX* | -0.018 | 0.119 | -0.150 | 0.883 | -0.277 | 0.241 |
| *TP53* | -0.009 | 0.111 | -0.080 | 0.938 | -0.250 | 0.232 |
| *MGMT* | -0.005 | 0.086 | -0.063 | 0.951 | -0.192 | 0.182 |
| *TERT* | 0.031 | 0.082 | 0.375 | 0.714 | -0.147 | 0.209 |
| *1p/19q* | -0.110 | 0.100 | -1.097 | 0.294 | -0.329 | 0.109 |
| WHO grade^a^ | -0.086 | 0.093 | -0.925 | 0.373 | -0.288 | 0.116 |
| Oligo./Astro.^b^ | -0.172 | 0.155 | -1.106 | 0.290 | -0.510 | 0.166 |
| Ki-67^c^ | -0.035 | 0.102 | -0.341 | 0.739 | -0.257 | 0.188 |

**Abbreviation:** IRE: insular glioma related epilepsy; tumors located on the left, IRE_L; coef: Coefficient; std err: Standard Error; t: t value; *p*: *p* value; CI: Confidence Interval; IDH: Isocitrate Dehydrogenase; ATRX: Alpha Thalassemia/Mental Retardation Syndrome X-linked; TP53: Tumor Protein 53; MGMT: O-6 Methylguanine-DNA Methyltransferase; TERT: Telomerase Reverse Transcriptase; 1p/19q: 1p/19q Chromosome Codeletion; WHO: World Health Organization; Oligo./Astro. : Oligodendroglioma or Astrocytoma. **The detail was not explained ensured the table was clear.** ^a^ Patients were divided into low- and high grade subgoups. ^b^ Patients were divided into Oligo./Astro. and other histopathological subtypes. ^c^ Patients were divide into Ki-67 < 10% and Ki-67 > 10% subgroups.
