# Supplementary material for: The Dual Associations of Peripheral Inflammatory Cells With Brain Reorganization in Insular Gliomas With/Without Epilepsy: An Exploratory Analysis
Source: CNS Neurosci Ther. 2026 Feb 20;32(2):e70788. doi: 10.1002/cns.70788 (PMC12927981; doi:10.1002/cns.70788)
Supplement: Supplementary file 10 — Table S4: Multivariable regression analysis of brain reorganization in the medial inferior temporal gray matter volume of IRE_R and clinical variables. [file CNS-32-e70788-s019.docx]

**Table S4. Multivariable regression analysis of brain reorganization in the medial inferior temporal grey matter volume of IRE_R and clinical variables.**

| Variables | coef. | std. err. | t | *p* > \|t\| | 95% CI  Lower | 95% CI Upper |
| --- | --- | --- | --- | --- | --- | --- |
| Gender | -0.067 | 0.066 | -1.008 | 0.335 | -0.212 | 0.079 |
| Age | 0.002 | 0.003 | 0.672 | 0.516 | -0.004 | 0.008 |
| Time of duration | 0 | 0 | 0.136 | 0.894 | 0 | 0 |
| Tumor volume | 0 | 0 | -0.708 | 0.494 | 0 | 0 |
| *IDH* | 0.152 | 0.138 | 1.096 | 0.297 | -0.153 | 0.456 |
| *ATRX* | 0.042 | 0.042 | 0.999 | 0.339 | -0.051 | 0.135 |
| *TP53* | 0.006 | 0.043 | 0.14 | 0.891 | -0.09 | 0.102 |
| *MGMT* | 0.0125 | 0.056 | 0.224 | 0.827 | -0.111 | 0.136 |
| *TERT* | -0.005 | 0.045 | -0.102 | 0.921 | -0.103 | 0.094 |
| *1p/19q* | 0.020 | 0.035 | 0.574 | 0.578 | -0.058 | 0.098 |
| WHO grade^a^ | -0.044 | 0.047 | -0.942 | 0.366 | -0.148 | 0.059 |
| Oligo./Astro.^b^ | -0.085 | 0.207 | -0.412 | 0.688 | -0.542 | 0.371 |
| Ki-67^c^ | 0.088 | 0.069 | 1.28 | 0.227 | -0.064 | 0.240 |

**Abbreviation:** IRE: insular glioma related epilepsy; tumors located on the right, IRE_R; coef: Coefficient; std err: Standard Error; t: t value; *p*: *p* value; CI: Confidence Interval; IDH: Isocitrate Dehydrogenase; ATRX: Alpha Thalassemia/Mental Retardation Syndrome X-linked; TP53: Tumor Protein 53; MGMT: O-6 Methylguanine-DNA Methyltransferase; TERT: Telomerase Reverse Transcriptase; 1p/19q: 1p/19q Chromosome Codeletion; WHO: World Health Organization; Oligo./Astro. : Oligodendroglioma or Astrocytoma. **The detail was not explained ensured the table was clear.** ^a^ Patients were divided into low- and high grade subgoups. ^b^ Patients were divided into Oligo./Astro. and other histopathological subtypes. ^c^ Patients were divided into Ki-67 < 10% and Ki-67 > 10% subgoups.
