# Supplementary material for: The Dual Associations of Peripheral Inflammatory Cells With Brain Reorganization in Insular Gliomas With/Without Epilepsy: An Exploratory Analysis
Source: CNS Neurosci Ther. 2026 Feb 20;32(2):e70788. doi: 10.1002/cns.70788 (PMC12927981; doi:10.1002/cns.70788)
Supplement: Supplementary file 12 — Table S6: Multivariable regression analysis of brain reorganization in the superior frontal cortex of IRE_R and clinical variables. [file CNS-32-e70788-s013.docx]

**Table S6. Multivariable regression analysis of brain reorganization in the superior frontal cortex of IRE_R and clinical variables.**

| Variables | coef. | std. err. | t | *p* > \|t\| | 95% CI  Lower | 95% CI Upper |
| --- | --- | --- | --- | --- | --- | --- |
| Gender | 0.006 | 0.008 | 0.697 | 0.500 | -0.012 | 0.024 |
| Age | 0 | 0 | -0.375 | 0.715 | -0.001 | 0.001 |
| Time of duration | 0 | 0 | -0.156 | 0.879 | 0 | 0 |
| Tumor volume | 0 | 0 | -0.745 | 0.472 | 0 | 0 |
| *IDH* | 0.005 | 0.017 | 0.291 | 0.777 | -0.033 | 0.042 |
| *ATRX* | 0.003 | 0.005 | 0.657 | 0.525 | -0.008 | 0.015 |
| *TP53* | -0.003 | 0.005 | -0.610 | 0.554 | -0.015 | 0.009 |
| *MGMT* | -0.002 | 0.007 | -0.259 | 0.800 | -0.017 | 0.013 |
| *TERT* | 0.004 | 0.005 | 0.702 | 0.497 | -0.008 | 0.016 |
| *1p/19q* | -0.001 | 0.004 | -0.273 | 0.790 | -0.011 | 0.008 |
| WHO grade^a^ | -0.007 | 0.006 | -1.184 | 0.261 | -0.020 | 0.006 |
| Oligo./Astro.^b^ | -0.005 | 0.026 | -0.207 | 0.840 | -0.061 | 0.051 |
| Ki-67^c^ | 0.012 | 0.008 | 1.368 | 0.198 | -0.007 | 0.030 |

**Abbreviation:** IRE: insular glioma related epilepsy; tumors located on the right, IRE_R; coef: Coefficient; std err: Standard Error; t: t value; *p*: *p* value; CI: Confidence Interval; IDH: Isocitrate Dehydrogenase; ATRX: Alpha Thalassemia/Mental Retardation Syndrome X-linked; TP53: Tumor Protein 53; MGMT: O-6 Methylguanine-DNA Methyltransferase; TERT: Telomerase Reverse Transcriptase; 1p/19q: 1p/19q Chromosome Codeletion; WHO: World Health Organization; Oligo./Astro. : Oligodendroglioma or Astrocytoma. **The detail was not explained ensured the table was clear.** ^a^ Patients were divided into low- and high grade subgoups. ^b^ Patients were divided into Oligo./Astro. and other histopathological subtypes. ^c^ Patients were divided into Ki-67 < 10% and Ki-67 > 10% subgroups.
