# Supplementary material for: The Dual Associations of Peripheral Inflammatory Cells With Brain Reorganization in Insular Gliomas With/Without Epilepsy: An Exploratory Analysis
Source: CNS Neurosci Ther. 2026 Feb 20;32(2):e70788. doi: 10.1002/cns.70788 (PMC12927981; doi:10.1002/cns.70788)
Supplement: Supplementary file 14 — Table S8: Multivariable regression analysis of brain reorganization in the precentral cortex of IRE_R and clinical variables. [file CNS-32-e70788-s017.docx]

**Table S8. Multivariable regression analysis of brain reorganization in the precentral cortex of IRE_R and clinical variables.**

| Variables | coef. | std. err. | t | *p* > \|t\| | 95% CI  Lower | 95% CI Upper |
| --- | --- | --- | --- | --- | --- | --- |
| Gender | -0.014 | 0.013 | -1.078 | 0.304 | -0.043 | 0.015 |
| Age | 0 | 0.001 | 0.507 | 0.622 | -0.001 | 0.001 |
| Time of duration | 0 | 0 | -1.638 | 0.130 | 0 | 0 |
| Tumor volume | 0 | 0 | 1.025 | 0.328 | 0 | 0 |
| *IDH* | -0.023 | 0.027 | -0.850 | 0.414 | -0.084 | 0.037 |
| *ATRX* | 0.013 | 0.008 | 1.504 | 0.161 | -0.006 | 0.031 |
| *TP53* | -0.009 | 0.009 | -1.034 | 0.323 | -0.028 | 0.010 |
| *MGMT* | -0.010 | 0.011 | -0.890 | 0.392 | -0.034 | 0.015 |
| *TERT* | 0.023 | 0.009 | 2.631 | 0.023 | 0.004 | 0.043 |
| *1p/19q* | 0.001 | 0.007 | 0.139 | 0.892 | -0.015 | 0.016 |
| WHO grade^a^ | -0.007 | 0.009 | -0.696 | 0.501 | -0.027 | 0.014 |
| Oligo./Astro.^b^ | 0.049 | 0.041 | 1.187 | 0.260 | -0.042 | 0.140 |
| Ki-67^c^ | 0.046 | 0.014 | 3.381 | 0.006 | 0.016 | 0.077 |

**Abbreviation:** IRE: insular glioma related epilepsy; tumors located on the right, IRE_R; coef: Coefficient; std err: Standard Error; t: t value; *p*: *p* value; CI: Confidence Interval; IDH: Isocitrate Dehydrogenase; ATRX: Alpha Thalassemia/Mental Retardation Syndrome X-linked; TP53: Tumor Protein 53; MGMT: O-6 Methylguanine-DNA Methyltransferase; TERT: Telomerase Reverse Transcriptase; 1p/19q: 1p/19q Chromosome Codeletion; WHO: World Health Organization; Oligo./Astro. : Oligodendroglioma or Astrocytoma. **The detail was not explained ensured the table was clear.** ^a^ Patients were divided into low- and high grade subgoups. ^b^ Patients were divided into Oligo./Astro. and other histopathological subtypes. ^c^ Patients were divided into Ki-67 < 10% and Ki-67 > 10% subgroups.
