# Supplementary material for: The Dual Associations of Peripheral Inflammatory Cells With Brain Reorganization in Insular Gliomas With/Without Epilepsy: An Exploratory Analysis
Source: CNS Neurosci Ther. 2026 Feb 20;32(2):e70788. doi: 10.1002/cns.70788 (PMC12927981; doi:10.1002/cns.70788)
Supplement: Supplementary file 15 — Table S9: Multivariable regression analysis of brain reorganization after principal component analysis of IRE_R and clinical variables. [file CNS-32-e70788-s003.docx]

**Table S9. Multivariable regression analysis of brain reorganization after principal component analysis of IRE_R and clinical variables.**

| Variables | coef. | std. err. | t | *p* > \|t\| | 95% CI  Lower | 95% CI Upper |
| --- | --- | --- | --- | --- | --- | --- |
| Gender | 0 | 0.015 | -0.011 | 0.991 | -0.034 | 0.033 |
| Age | 0 | 0.001 | -0.696 | 0.501 | -0.002 | 0.001 |
| Time of duration | 0 | 0 | 1.079 | 0.304 | 0 | 0 |
| Tumor volume | 0 | 0 | -1.020 | 0.330 | 0 | 0 |
| *IDH* | 0.025 | 0.032 | 0.776 | 0.454 | -0.045 | 0.095 |
| *ATRX* | 0.004 | 0.010 | 0.424 | 0.680 | -0.017 | 0.026 |
| *TP53* | 0.003 | 0.010 | 0.258 | 0.801 | -0.019 | 0.025 |
| *MGMT* | 0.009 | 0.013 | 0.663 | 0.521 | -0.020 | 0.037 |
| *TERT* | -0.011 | 0.010 | -1.090 | 0.299 | -0.034 | 0.011 |
| *1p/19q* | 0.004 | 0.008 | 0.454 | 0.659 | -0.014 | 0.022 |
| WHO grade^a^ | -0.007 | 0.011 | -0.678 | 0.512 | -0.031 | 0.016 |
| Oligo./Astro.^b^ | -0.047 | 0.048 | -0.981 | 0.348 | -0.152 | 0.058 |
| Ki-67^c^ | -0.007 | 0.016 | -0.450 | 0.661 | -0.042 | 0.028 |

**Abbreviation:** IRE: insular glioma related epilepsy; tumors located on the right, IRE_R; coef: Coefficient; std err: Standard Error; t: t value; *p*: *p* value; CI: Confidence Interval; IDH: Isocitrate Dehydrogenase; ATRX: Alpha Thalassemia/Mental Retardation Syndrome X-linked; TP53: Tumor Protein 53; MGMT: O-6 Methylguanine-DNA Methyltransferase; TERT: Telomerase Reverse Transcriptase; 1p/19q: 1p/19q Chromosome Codeletion; WHO: World Health Organization; Oligo./Astro. : Oligodendroglioma or Astrocytoma. **The detail was not explained ensured the table was clear.** ^a^ Patients were divided into low- and high grade subgoups. ^b^ Patients were divided into Oligo./Astro. and other histopathological subtypes. ^c^ Patients were divided into Ki-67 < 10% and Ki-67 > 10% subgroups.
