# Supplementary material for: The Dual Associations of Peripheral Inflammatory Cells With Brain Reorganization in Insular Gliomas With/Without Epilepsy: An Exploratory Analysis
Source: CNS Neurosci Ther. 2026 Feb 20;32(2):e70788. doi: 10.1002/cns.70788 (PMC12927981; doi:10.1002/cns.70788)
Supplement: Supplementary file 16 — Table S10: Multivariable regression analysis of brain reorganization in the superior frontal cortex of IRnE_L and clinical variables. [file CNS-32-e70788-s011.docx]

**Table S10. Multivariable regression analysis of brain reorganization in the superior frontal cortex of IRnE_L and clinical variables.**

| Variables | coef. | std. err. | t | *p* > \|t\| | 95% CI  Lower | 95% CI Upper |
| --- | --- | --- | --- | --- | --- | --- |
| Gender | -1.501 | 0.736 | -2.039 | 0.066 | -3.122 | 0.120 |
| Age | -0.004 | 0.023 | -0.150 | 0.884 | -0.055 | 0.048 |
| Time of duration | 0.005 | 0.004 | 1.266 | 0.232 | -0.003 | 0.012 |
| Tumor volume | 0 | 0 | -0.836 | 0.421 | 0 | 0 |
| *IDH* | -1.322 | 0.702 | -1.885 | 0.086 | -2.867 | 0.222 |
| *ATRX* | -0.401 | 0.492 | -0.815 | 0.432 | -1.484 | 0.682 |
| *TP53* | 0.761 | 0.681 | 1.118 | 0.287 | -0.737 | 2.260 |
| *MGMT* | -0.479 | 0.869 | -0.551 | 0.593 | -2.391 | 1.434 |
| *TERT* | -0.181 | 0.530 | -0.342 | 0.739 | -1.348 | 0.986 |
| *1p/19q* | -0.539 | 0.337 | -1.603 | 0.137 | -1.280 | 0.201 |
| WHO grade^a^ | -0.086 | 0.914 | -0.094 | 0.926 | -2.099 | 1.926 |
| Oligo./Astro.^b^ | 0.333 | 0.936 | 0.356 | 0.729 | -1.728 | 2.394 |
| Ki-67^c^ | -0.162 | 0.803 | -0.202 | 0.844 | -1.928 | 1.604 |

**Abbreviation:** IRnE: insular glioma without epilepsy; tumors located on the left, IRnE_L; coef: Coefficient; std err: Standard Error; t: t value; *p*: *p* value; CI: Confidence Interval; IDH: Isocitrate Dehydrogenase; ATRX: Alpha Thalassemia/Mental Retardation Syndrome X-linked; TP53: Tumor Protein 53; MGMT: O-6 Methylguanine-DNA Methyltransferase; TERT: Telomerase Reverse Transcriptase; 1p/19q: 1p/19q Chromosome Codeletion; WHO: World Health Organization; Oligo./Astro. : Oligodendroglioma or Astrocytoma. **The detail was not explained ensured the table was clear.** ^a^ Patients were divided into low- and high grade subgoups. ^b^ Patients were divided into Oligo./Astro. and other histopathological subtypes. ^c^ Patients were divided into Ki-67 < 10% and Ki-67 > 10% subgroups.
