# Supplementary material for: The Dual Associations of Peripheral Inflammatory Cells With Brain Reorganization in Insular Gliomas With/Without Epilepsy: An Exploratory Analysis
Source: CNS Neurosci Ther. 2026 Feb 20;32(2):e70788. doi: 10.1002/cns.70788 (PMC12927981; doi:10.1002/cns.70788)
Supplement: Supplementary file 17 — Table S11: Multivariable regression analysis of brain reorganization in the middle frontal cortex of IRnE_L and clinical variables. [file CNS-32-e70788-s009.docx]

**Table S11. Multivariable regression analysis of brain reorganization in the middle frontal cortex of IRnE_L and clinical variables.**

| Variables | coef. | std. err. | t | *p* > \|t\| | 95% CI  Lower | 95% CI Upper |
| --- | --- | --- | --- | --- | --- | --- |
| Gender | -0.690 | 0.716 | -0.964 | 0.356 | -2.265 | 0.885 |
| Age | 0.057 | 0.031 | 1.832 | 0.094 | -0.011 | 0.125 |
| Time of duration | -0.002 | 0.005 | -0.360 | 0.726 | -0.013 | 0.009 |
| Tumor volume | 0 | 0 | -1.212 | 0.251 | 0 | 0 |
| *IDH* | 0.641 | 0.872 | 0.735 | 0.477 | -1.278 | 2.560 |
| *ATRX* | -0.856 | 0.644 | -1.329 | 0.211 | -2.274 | 0.562 |
| *TP53* | 1.015 | 0.899 | 1.129 | 0.283 | -0.965 | 2.995 |
| *MGMT* | 1.912 | 1.114 | 1.716 | 0.114 | -0.540 | 4.363 |
| *TERT* | -0.886 | 0.715 | -1.239 | 0.241 | -2.460 | 0.688 |
| *1p/19q* | -0.268 | 0.442 | -0.607 | 0.556 | -1.241 | 0.704 |
| WHO grade^a^ | 0.186 | 1.162 | 0.160 | 0.876 | -2.372 | 2.744 |
| Oligo./Astro.^b^ | 1.385 | 1.317 | 1.052 | 0.316 | -1.514 | 4.284 |
| Ki-67^c^ | 0.471 | 1.041 | 0.453 | 0.660 | -1.821 | 2.764 |

**Abbreviation:** IRnE: insular glioma without epilepsy; tumors located on the left, IRnE_L; coef: Coefficient; std err: Standard Error; t: t value; *p*: *p* value; CI: Confidence Interval; IDH: Isocitrate Dehydrogenase; ATRX: Alpha Thalassemia/Mental Retardation Syndrome X-linked; TP53: Tumor Protein 53; MGMT: O-6 Methylguanine-DNA Methyltransferase; TERT: Telomerase Reverse Transcriptase; 1p/19q: 1p/19q Chromosome Codeletion; WHO: World Health Organization; Oligo./Astro. : Oligodendroglioma or Astrocytoma. **The detail was not explained ensured the table was clear.** ^a^ Patients were divided into low- and high grade subgoups. ^b^ Patients were divided into Oligo./Astro. and other histopathological subtypes. ^c^ Patients were divided into Ki-67 < 10% and Ki-67 > 10% subgroups.
