# Supplementary material for: The Dual Associations of Peripheral Inflammatory Cells With Brain Reorganization in Insular Gliomas With/Without Epilepsy: An Exploratory Analysis
Source: CNS Neurosci Ther. 2026 Feb 20;32(2):e70788. doi: 10.1002/cns.70788 (PMC12927981; doi:10.1002/cns.70788)
Supplement: Supplementary file 18 — Table S12: Multivariable regression analysis of brain reorganization in the middle temporal cortex of IRnE_L and clinical variables. [file CNS-32-e70788-s025.docx]

**Table S12. Multivariable regression analysis of brain reorganization in the middle temporal cortex of IRnE_L and clinical variables.**

| Variables | coef. | std. err. | t | *p* > \|t\| | 95% CI  Lower | 95% CI Upper |
| --- | --- | --- | --- | --- | --- | --- |
| Gender | 0.471 | 0.643 | 0.733 | 0.479 | -0.944 | 1.885 |
| Age | 0.017 | 0.028 | 0.620 | 0.548 | -0.044 | 0.079 |
| Time of duration | 0.003 | 0.005 | 0.670 | 0.517 | -0.007 | 0.013 |
| Tumor volume | 0 | 0 | -0.200 | 0.845 | 0 | 0 |
| *IDH* | -0.188 | 0.783 | -0.241 | 0.814 | -1.912 | 1.535 |
| *ATRX* | -0.167 | 0.579 | -0.288 | 0.779 | -1.440 | 1.107 |
| *TP53* | 0.006 | 0.808 | 0.008 | 0.994 | -1.771 | 1.784 |
| *MGMT* | -0.404 | 1.000 | -0.404 | 0.694 | -2.605 | 1.798 |
| *TERT* | -0.471 | 0.642 | -0.733 | 0.479 | -1.884 | 0.942 |
| *1p/19q* | 0.436 | 0.397 | 1.098 | 0.296 | -0.438 | 1.309 |
| WHO grade^a^ | 2.130 | 1.044 | 2.042 | 0.066 | -0.166 | 4.427 |
| Oligo./Astro.^b^ | 0.644 | 1.183 | 0.544 | 0.597 | -1.959 | 3.247 |
| Ki-67^c^ | -2.237 | 0.935 | -2.392 | 0.036 | -4.295 | -0.179 |

**Abbreviation:** IRnE: insular glioma without epilepsy; tumors located on the left, IRnE_L; coef: Coefficient; std err: Standard Error; t: t value; *p*: *p* value; CI: Confidence Interval; IDH: Isocitrate Dehydrogenase; ATRX: Alpha Thalassemia/Mental Retardation Syndrome X-linked; TP53: Tumor Protein 53; MGMT: O-6 Methylguanine-DNA Methyltransferase; TERT: Telomerase Reverse Transcriptase; 1p/19q: 1p/19q Chromosome Codeletion; WHO: World Health Organization; Oligo./Astro. : Oligodendroglioma or Astrocytoma. **The detail was not explained ensured the table was clear.** ^a^ Patients were divided into low- and high grade subgoups. ^b^ Patients were divided into Oligo./Astro. and other histopathological subtypes. ^c^ Patients were divided into Ki-67 < 10% and Ki-67 > 10% subgroups.
