# Supplementary material for: The Dual Associations of Peripheral Inflammatory Cells With Brain Reorganization in Insular Gliomas With/Without Epilepsy: An Exploratory Analysis
Source: CNS Neurosci Ther. 2026 Feb 20;32(2):e70788. doi: 10.1002/cns.70788 (PMC12927981; doi:10.1002/cns.70788)
Supplement: Supplementary file 19 — Table S13: Multivariable regression analysis of brain reorganization in the inferior temporal cortex of IRnE_L and clinical variables. [file CNS-32-e70788-s022.docx]

**Table S13. Multivariable regression analysis of brain reorganization in the inferior temporal cortex of IRnE_L and clinical variables.**

| Variables | coef. | std. err. | t | *p* > \|t\| | 95% CI  Lower | 95% CI Upper |
| --- | --- | --- | --- | --- | --- | --- |
| Gender | 0.198 | 0.512 | 0.388 | 0.706 | -0.927 | 1.324 |
| Age | -0.010 | 0.022 | -0.458 | 0.656 | -0.059 | 0.039 |
| Time of duration | 0.004 | 0.004 | 0.979 | 0.349 | -0.004 | 0.011 |
| Tumor volume | 0 | 0 | -1.293 | 0.222 | 0 | 0 |
| *IDH* | 0.424 | 0.623 | 0.681 | 0.510 | -0.947 | 1.796 |
| *ATRX* | -0.024 | 0.460 | -0.052 | 0.960 | -1.037 | 0.990 |
| *TP53* | 0.540 | 0.643 | 0.840 | 0.419 | -0.875 | 1.955 |
| *MGMT* | -1.231 | 0.796 | -1.547 | 0.150 | -2.983 | 0.521 |
| *TERT* | 0.134 | 0.511 | 0.263 | 0.798 | -0.991 | 1.259 |
| *1p/19q* | 0.001 | 0.316 | 0.003 | 0.998 | -0.694 | 0.696 |
| WHO grade^a^ | 1.260 | 0.831 | 1.517 | 0.157 | -0.568 | 3.088 |
| Oligo./Astro.^b^ | 0.377 | 0.941 | 0.400 | 0.697 | -1.695 | 2.449 |
| Ki-67^c^ | -1.363 | 0.744 | -1.831 | 0.094 | -3.001 | 0.275 |

**Abbreviation:** IRnE: insular glioma without epilepsy; tumors located on the left, IRnE_L; coef: Coefficient; std err: Standard Error; t: t value; *p*: *p* value; CI: Confidence Interval; IDH: Isocitrate Dehydrogenase; ATRX: Alpha Thalassemia/Mental Retardation Syndrome X-linked; TP53: Tumor Protein 53; MGMT: O-6 Methylguanine-DNA Methyltransferase; TERT: Telomerase Reverse Transcriptase; 1p/19q: 1p/19q Chromosome Codeletion; WHO: World Health Organization; Oligo./Astro. : Oligodendroglioma or Astrocytoma. **The detail was not explained ensured the table was clear.** ^a^ Patients were divided into low- and high grade subgoups. ^b^ Patients were divided into Oligo./Astro. and other histopathological subtypes. ^c^ Patients were divided into Ki-67 < 10% and Ki-67 > 10% subgroups.
