# Supplementary material for: The Dual Associations of Peripheral Inflammatory Cells With Brain Reorganization in Insular Gliomas With/Without Epilepsy: An Exploratory Analysis
Source: CNS Neurosci Ther. 2026 Feb 20;32(2):e70788. doi: 10.1002/cns.70788 (PMC12927981; doi:10.1002/cns.70788)
Supplement: Supplementary file 20 — Table S14: Multivariable regression analysis of brain reorganization in the precuneus cortex of IRnE_L and clinical variables. [file CNS-32-e70788-s016.docx]

**Table S14. Multivariable regression analysis of brain reorganization in the precuneus cortex of IRnE_L and clinical variables.**

| Variables | coef. | std. err. | t | *p* > \|t\| | 95% CI  Lower | 95% CI Upper |
| --- | --- | --- | --- | --- | --- | --- |
| Gender | 0.490 | 0.557 | 0.879 | 0.398 | -0.737 | 1.716 |
| Age | 0.015 | 0.024 | 0.609 | 0.555 | -0.038 | 0.067 |
| Time of duration | 0.008 | 0.004 | 2.109 | 0.059 | 0 | 0.016 |
| Tumor volume | 0 | 0 | -1.103 | 0.294 | 0 | 0 |
| *IDH* | 0.310 | 0.672 | 0.461 | 0.654 | -1.170 | 1.790 |
| *ATRX* | -0.150 | 0.509 | -0.295 | 0.774 | -1.271 | 0.971 |
| *TP53* | 0.763 | 0.664 | 1.150 | 0.275 | -0.698 | 2.225 |
| *MGMT* | -0.787 | 0.438 | -1.799 | 0.100 | -1.751 | 0.176 |
| *TERT* | -0.637 | 0.358 | -1.782 | 0.102 | -1.425 | 0.150 |
| *1p/19q* | 0.077 | 0.372 | 0.206 | 0.840 | -0.742 | 0.896 |
| WHO grade^a^ | 0.741 | 0.774 | 0.957 | 0.359 | -0.962 | 2.444 |
| Oligo./Astro.^b^ | -0.112 | 0.970 | -0.116 | 0.910 | -2.246 | 2.022 |
| Ki-67^c^ | -0.731 | 0.752 | -0.972 | 0.352 | -2.385 | 0.924 |

**Abbreviation:** IRnE: insular glioma without epilepsy; tumors located on the left, IRnE_L; coef: Coefficient; std err: Standard Error; t: t value; *p*: *p* value; CI: Confidence Interval; IDH: Isocitrate Dehydrogenase; ATRX: Alpha Thalassemia/Mental Retardation Syndrome X-linked; TP53: Tumor Protein 53; MGMT: O-6 Methylguanine-DNA Methyltransferase; TERT: Telomerase Reverse Transcriptase; 1p/19q: 1p/19q Chromosome Codeletion; WHO: World Health Organization; Oligo./Astro. : Oligodendroglioma or Astrocytoma. **The detail was not explained ensured the table was clear.** ^a^ Patients were divided into low- and high grade subgoups. ^b^ Patients were divided into Oligo./Astro. and other histopathological subtypes. ^c^ Patients were divided into Ki-67 < 10% and Ki-67 > 10% subgroups.
