# Supplementary material for: The Dual Associations of Peripheral Inflammatory Cells With Brain Reorganization in Insular Gliomas With/Without Epilepsy: An Exploratory Analysis
Source: CNS Neurosci Ther. 2026 Feb 20;32(2):e70788. doi: 10.1002/cns.70788 (PMC12927981; doi:10.1002/cns.70788)
Supplement: Supplementary file 21 — Table S15: Multivariable regression analysis of brain reorganization after principal component analysis of IRE_L and clinical variables. [file CNS-32-e70788-s028.docx]

**Table S15. Multivariable regression analysis of brain reorganization after principal component analysis of IRE_L and clinical variables.**

| Variables | coef. | std. err. | t | *p* > \|t\| | 95% CI  Lower | 95% CI Upper |
| --- | --- | --- | --- | --- | --- | --- |
| Gender | 0.326 | 0.571 | 0.570 | 0.580 | -0.932 | 1.583 |
| Age | 0.009 | 0.024 | 0.387 | 0.706 | -0.043 | 0.062 |
| Time of duration | -0.001 | 0.001 | -0.926 | 0.374 | -0.003 | 0.001 |
| Tumor volume | 0 | 0 | 0.572 | 0.579 | 0 | 0 |
| *IDH* | -0.516 | 1.195 | -0.432 | 0.674 | -3.146 | 2.114 |
| *ATRX* | 0.253 | 0.365 | 0.692 | 0.503 | -0.551 | 1.056 |
| *TP53* | -0.527 | 0.376 | -1.404 | 0.188 | -1.354 | 0.299 |
| *MGMT* | -0.351 | 0.483 | -0.728 | 0.482 | -1.414 | 0.711 |
| *TERT* | 0.077 | 0.386 | 0.198 | 0.846 | -0.772 | 0.925 |
| *1p/19q* | -0.021 | 0.306 | -0.069 | 0.946 | -0.694 | 0.652 |
| WHO grade^a^ | 0.207 | 0.405 | 0.510 | 0.620 | -0.685 | 1.098 |
| Oligo./Astro.^b^ | 1.088 | 1.791 | 0.607 | 0.556 | -2.854 | 5.029 |
| Ki-67^c^ | 0.243 | 0.596 | 0.408 | 0.691 | -1.068 | 1.554 |

**Abbreviation:** IRnE: insular glioma without epilepsy; tumors located on the left, IRnE_L; coef: Coefficient; std err: Standard Error; t: t value; *p*: *p* value; CI: Confidence Interval; IDH: Isocitrate Dehydrogenase; ATRX: Alpha Thalassemia/Mental Retardation Syndrome X-linked; TP53: Tumor Protein 53; MGMT: O-6 Methylguanine-DNA Methyltransferase; TERT: Telomerase Reverse Transcriptase; 1p/19q: 1p/19q Chromosome Codeletion; WHO: World Health Organization; Oligo./Astro. : Oligodendroglioma or Astrocytoma. **The detail was not explained ensured the table was clear.** ^a^ Patients were divided into low- and high grade subgoups. ^b^ Patients were divided into Oligo./Astro. and other histopathological subtypes. ^c^ Patients were divided into Ki-67 < 10% and Ki-67 > 10% subgroups.
