# Supplementary material for: The Dual Associations of Peripheral Inflammatory Cells With Brain Reorganization in Insular Gliomas With/Without Epilepsy: An Exploratory Analysis
Source: CNS Neurosci Ther. 2026 Feb 20;32(2):e70788. doi: 10.1002/cns.70788 (PMC12927981; doi:10.1002/cns.70788)
Supplement: Supplementary file 22 — Table S16: Multivariable regression analysis of brain reorganization in the inferior temporal cortex of IRnE_R and clinical variables. [file CNS-32-e70788-s008.docx]

**Table S16. Multivariable regression analysis of brain reorganization in the inferior temporal cortex of IRnE_R and clinical variables.**

| Variables | coef. | std. err. | t | *p* > \|t\| | 95% CI  Lower | 95% CI Upper |
| --- | --- | --- | --- | --- | --- | --- |
| Gender | 0.995 | 0.511 | 1.949 | 0.072 | -0.100 | 2.091 |
| Age | 0 | 0.018 | 0.024 | 0.981 | -0.039 | 0.040 |
| Time of duration | -0.002 | 0.001 | -1.381 | 0.189 | -0.005 | 0.001 |
| Tumor volume | 0 | 0 | 1.614 | 0.129 | 0 | 0 |
| *IDH* | -0.837 | 0.459 | -1.821 | 0.090 | -1.822 | 0.149 |
| *ATRX* | -0.479 | 0.644 | -0.744 | 0.469 | -1.859 | 0.901 |
| *TP53* | 0.889 | 0.770 | 1.155 | 0.267 | -0.762 | 2.540 |
| *MGMT* | -0.392 | 0.620 | -0.632 | 0.537 | -1.722 | 0.938 |
| *TERT* | -0.030 | 0.521 | -0.056 | 0.956 | -1.148 | 1.089 |
| *1p/19q* | -0.524 | 0.417 | -1.254 | 0.230 | -1.419 | 0.372 |
| WHO grade^a^ | 0.089 | 0.693 | 0.129 | 0.899 | -1.396 | 1.575 |
| Oligo./Astro.^b^ | -0.837 | 0.459 | -1.821 | 0.090 | -1.822 | 0.149 |
| Ki-67^c^ | -0.750 | 1.125 | -0.666 | 0.516 | -3.163 | 1.664 |

**Abbreviation:** IRnE: insular glioma without epilepsy; tumors located on the right, IRnE_R; coef: Coefficient; std err: Standard Error; t: t value; *p*: *p* value; CI: Confidence Interval; IDH: Isocitrate Dehydrogenase; ATRX: Alpha Thalassemia/Mental Retardation Syndrome X-linked; TP53: Tumor Protein 53; MGMT: O-6 Methylguanine-DNA Methyltransferase; TERT: Telomerase Reverse Transcriptase; 1p/19q: 1p/19q Chromosome Codeletion; WHO: World Health Organization; Oligo./Astro. : Oligodendroglioma or Astrocytoma. **The detail was not explained ensured the table was clear.** ^a^ Patients were divided into low- and high grade subgoups. ^b^ Patients were divided into Oligo./Astro. and other histopathological subtypes. ^c^ Patients were divided into Ki-67 < 10% and Ki-67 > 10% subgroups.
