# Supplementary material for: The Dual Associations of Peripheral Inflammatory Cells With Brain Reorganization in Insular Gliomas With/Without Epilepsy: An Exploratory Analysis
Source: CNS Neurosci Ther. 2026 Feb 20;32(2):e70788. doi: 10.1002/cns.70788 (PMC12927981; doi:10.1002/cns.70788)
Supplement: Supplementary file 23 — Table S17: Multivariable regression analysis of brain reorganization in the posterior cingulate cortex of IRnE_R and clinical variables. [file CNS-32-e70788-s002.docx]

**Table S17. Multivariable regression analysis of brain reorganization in the posterior cingulate cortex of IRnE_R and clinical variables.**

| Variables | coef. | std. err. | t | *p* > \|t\| | 95% CI  Lower | 95% CI Upper |
| --- | --- | --- | --- | --- | --- | --- |
| Gender | -1.104 | 0.601 | -1.839 | 0.087 | -2.392 | 0.184 |
| Age | -0.043 | 0.023 | -1.840 | 0.087 | -0.092 | 0.007 |
| Time of duration | -0.002 | 0.002 | -1.119 | 0.282 | -0.005 | 0.002 |
| Tumor volume | 0 | 0 | 1.195 | 0.252 | 0 | 0 |
| *IDH* | -0.037 | 0.548 | -0.067 | 0.948 | -1.212 | 1.139 |
| *ATRX* | 0.577 | 0.803 | 0.718 | 0.485 | -1.146 | 2.299 |
| *TP53* | -1.598 | 0.952 | -1.679 | 0.115 | -3.639 | 0.444 |
| *MGMT* | 0.855 | 0.774 | 1.103 | 0.288 | -0.806 | 2.516 |
| *TERT* | -0.129 | 0.660 | -0.195 | 0.848 | -1.545 | 1.287 |
| *1p/19q* | -0.405 | 0.523 | -0.775 | 0.451 | -1.527 | 0.717 |
| WHO grade^a^ | 0.764 | 0.872 | 0.877 | 0.395 | -1.105 | 2.634 |
| Oligo./Astro.^b^ | -0.037 | 0.548 | -0.067 | 0.948 | -1.212 | 1.139 |
| Ki-67^c^ | -1.787 | 1.386 | -1.290 | 0.218 | -4.759 | 1.184 |

**Abbreviation:** IRnE: insular glioma without epilepsy; tumors located on the right, IRnE_R; coef: Coefficient; std err: Standard Error; t: t value; *p*: *p* value; CI: Confidence Interval; IDH: Isocitrate Dehydrogenase; ATRX: Alpha Thalassemia/Mental Retardation Syndrome X-linked; TP53: Tumor Protein 53; MGMT: O-6 Methylguanine-DNA Methyltransferase; TERT: Telomerase Reverse Transcriptase; 1p/19q: 1p/19q Chromosome Codeletion; WHO: World Health Organization; Oligo./Astro. : Oligodendroglioma or Astrocytoma. **The detail was not explained ensured the table was clear.** ^a^ Patients were divided into low- and high grade subgoups. ^b^ Patients were divided into Oligo./Astro. and other histopathological subtypes. ^c^ Patients were divided into Ki-67 < 10% and Ki-67 > 10% subgroups.
