# Supplementary material for: The Dual Associations of Peripheral Inflammatory Cells With Brain Reorganization in Insular Gliomas With/Without Epilepsy: An Exploratory Analysis
Source: CNS Neurosci Ther. 2026 Feb 20;32(2):e70788. doi: 10.1002/cns.70788 (PMC12927981; doi:10.1002/cns.70788)
Supplement: Supplementary file 24 — Table S18: Multivariable regression analysis of brain reorganization after principal component analysis of IRE_R and clinical variables. [file CNS-32-e70788-s014.docx]

**Table S18. Multivariable regression analysis of brain reorganization after principal component analysis of IRE_R and clinical variables.**

| Variables | coef. | std. err. | t | *p* > \|t\| | 95% CI  Lower | 95% CI Upper |
| --- | --- | --- | --- | --- | --- | --- |
| Gender | -0.125 | 0.341 | -0.367 | 0.719 | -0.856 | 0.606 |
| Age | -0.027 | 0.013 | -2.087 | 0.056 | -0.055 | 0.001 |
| Time of duration | -0.002 | 0.001 | -1.823 | 0.090 | -0.004 | 0 |
| Tumor volume | 0 | 0 | 1.568 | 0.139 | 0 | 0 |
| *IDH* | -0.442 | 0.311 | -1.420 | 0.178 | -1.109 | 0.226 |
| *ATRX* | 0.170 | 0.456 | 0.374 | 0.714 | -0.807 | 1.148 |
| *TP53* | -0.790 | 0.540 | -1.463 | 0.166 | -1.949 | 0.369 |
| *MGMT* | 0.788 | 0.440 | 1.792 | 0.095 | -0.155 | 1.731 |
| *TERT* | -0.111 | 0.375 | -0.296 | 0.772 | -0.915 | 0.693 |
| *1p/19q* | -0.508 | 0.297 | -1.711 | 0.109 | -1.145 | 0.129 |
| WHO grade^a^ | 0.385 | 0.495 | 0.778 | 0.450 | -0.676 | 1.446 |
| Oligo./Astro.^b^ | -0.442 | 0.311 | -1.420 | 0.178 | -1.109 | 0.226 |
| Ki-67^c^ | -1.315 | 0.787 | -1.672 | 0.117 | -3.002 | 0.372 |

**Abbreviation:** IRnE: insular glioma without epilepsy; tumors located on the right, IRnE_R; coef: coefficient; std err: standard Error; t: t value; *p*: *p* value; CI: confidence Interval; IDH: Isocitrate Dehydrogenase; ATRX: Alpha Thalassemia/Mental Retardation Syndrome X-linked; TP53: Tumor Protein 53; MGMT: O-6 Methylguanine-DNA Methyltransferase; TERT: Telomerase Reverse Transcriptase; 1p/19q: 1p/19q Chromosome Codeletion; WHO: World Health Organization; Oligo./Astro. : Oligodendroglioma or Astrocytoma. **The detail was not explained ensured the table was clear.** ^a^ Patients were divided into low- and high grade subgoups. ^b^ Patients were divided into Oligo./Astro. and other histopathological subtypes. ^c^ Patients were divided into Ki-67 < 10% and Ki-67 > 10% subgroups.
