# Supplementary material for: The Dual Associations of Peripheral Inflammatory Cells With Brain Reorganization in Insular Gliomas With/Without Epilepsy: An Exploratory Analysis
Source: CNS Neurosci Ther. 2026 Feb 20;32(2):e70788. doi: 10.1002/cns.70788 (PMC12927981; doi:10.1002/cns.70788)
Supplement: Supplementary file 25 — Table S19: PCA derived compensation indices across different models. [file CNS-32-e70788-s007.docx]

**Table S19 PCA derived compensation indices across different models.**

| Combination  regions | Variables  included | PC1  variance | Major loadings | PC1 retained |
| --- | --- | --- | --- | --- |
| IRE_R | 3 | 94.66% | Superior frontal (0.58);  Middle frontal (0.58);  Precentral (0.57) | Yes |
| IRnE_L | 5 | 48.40% | Middle frontal (-0.53);  Precuneus (+0.52) | Yes |
| IRnE_R | 2 | 69.91% | Inferior temporal (0.71);  Posterior cingulate (-0.71) | Yes |
| IRE | 4 | 37.75% | Precentral (IRE_R: -0.72);  Middle frontal (IER_L; -0.58) | Yes |
| IRnE | 7 | 40% | Inferior temporal (IRnE_R; -0.54);  Posterior cingulate (IRnE_R: -0.51);  Precuneus (IRnE_L:-0.49);  Middle temporal (IRnE_L:-0.39) | Yes |

**Abbreviations:** PCA: principal component analysis; PC1: the first principal component; IRE: insular glioma related epilepsy; tumors located on the left, IRE_L; tumors located on the right, IRE_R; IRnE: insular glioma without epilepsy; tumors located on the left, IRnE_L; tumors located on the right, IRnE_R;
