# Supplementary material for: The Dual Associations of Peripheral Inflammatory Cells With Brain Reorganization in Insular Gliomas With/Without Epilepsy: An Exploratory Analysis
Source: CNS Neurosci Ther. 2026 Feb 20;32(2):e70788. doi: 10.1002/cns.70788 (PMC12927981; doi:10.1002/cns.70788)
Supplement: Supplementary file 26 — Table S20: Correlations between the brain reorganization and postoperative events in IRE and IRnE. [file CNS-32-e70788-s006.docx]

**Table S20. Correlations between the brain reorganization and postoperative events in IRE and IRnE**

| Group | Tumor side | Variables | Postoperative events | Correlation | *p* | Postoperative events | Correlation | *p* |
| --- | --- | --- | --- | --- | --- | --- | --- | --- |
| IRE | L | GMV | Epilepsy recurrence | 0.21 | 0.32 | Tumor  recurrence | 0.18 | 0.41 |
|  | R |  |  | -0.27 | 0.29 |  | 0.06 | 0.83 |
| IRnE | L | *GI* |  | 0.34 | 0.18 |  | 0.41 | 0.12 |
|  | R |  |  | -0.34 | 0.14 |  | -0.36 | 0.11 |

**Abbreviation:** IRE: insular glioma related epilepsy; IRnE: insular glioma without epilepsy; L: left; R: right; *p*: *p* value; GMV: Grey matter volume; *GI*: Gyrification. The analysis relied on Spearman correlation analysis. Correlation > 0 indicated a potential positive association. Correlation < 0 indicated a potential negative association.
