# Supplementary material for: The Dual Associations of Peripheral Inflammatory Cells With Brain Reorganization in Insular Gliomas With/Without Epilepsy: An Exploratory Analysis
Source: CNS Neurosci Ther. 2026 Feb 20;32(2):e70788. doi: 10.1002/cns.70788 (PMC12927981; doi:10.1002/cns.70788)
Supplement: Supplementary file 27 — Table S21: Correlation matrix of the brain compensation regions and potential biomarkers in peripheral blood. [file CNS-32-e70788-s015.docx]

**Table S21. Correlation matrix of the brain compensation regions and potential biomarkers in peripheral blood**

| Region | MO | HGB | MCV | MCH | MCHC | RDW | RDW-CV | PDW | MPV | P-LCR | PCT | HCT |
| --- | --- | --- | --- | --- | --- | --- | --- | --- | --- | --- | --- | --- |
| GMV_IRE_L | 0.11 | -0.22 | 0.051 | 0.091 | 0.122 | 0.191 | 0.061 | 0.036 | 0.091 | 0.272 | -0.236 | -0.225 |
| GMV_IRE_R | 0.12 | 0.031 | -0.188 | -0.079 | -0.111 | -0.14 | -0.043 | -0.043 | 0.074 | 0.012 | 0.217 | -0.075 |
| Inferiorfrontal | -0.10 | -0.27 | 0.21 | 0.25 | 0.19 | 0.08 | -0.06 | 0.10 | 0.08 | 0.01 | -0.27 | -0.28 |
| Superiorfrontal | 0.21 | -0.05 | 0.21 | 0.22 | 0.13 | -0.01 | -0.31 | 0.11 | -0.02 | -0.02 | -0.02 | -0.14 |
| Middlefrontal | 0.02 | 0.03 | 0.17 | 0.22 | 0.18 | 0.02 | -0.23 | 0.19 | 0.08 | 0.15 | -0.29 | -0.05 |
| Precentral | 0.37 | 0.37 | -0.06 | 0.24 | 0.05 | 0.21 | 0.25 | 0.04 | 0.30 | 0.15 | -0.04 | 0.16 |
| Superiorfrontal | -0.29 | 0.47* | -0.09 | -0.20 | -0.15 | -0.13 | 0.23 | 0.02 | -0.18 | -0.14 | -0.16 | 0.46* |
| Middlefrontal | -0.49* | 0.02 | 0.12 | 0.23 | 0.34 | -0.27 | -0.29 | 0.14 | -0.11 | -0.10 | -0.28 | 0.00 |
| Middletemporal | 0.09 | -0.01 | 0.15 | -0.08 | -0.30 | -0.02 | 0.19 | -0.03 | 0.02 | 0.06 | 0.02 | 0.03 |
| Inferiortemporal | -0.18 | 0.10 | -0.14 | -0.30 | -0.26 | -0.03 | 0.19 | 0.19 | 0.17 | 0.18 | 0.11 | 0.16 |
| Precuneus | -0.22 | -0.05 | 0.10 | -0.15 | -0.17 | -0.04 | -0.01 | 0.14 | -0.02 | 0.02 | -0.01 | -0.05 |
| InferiorTemporal | 0.00 | -0.61*** | 0.06 | -0.05 | -0.24 | 0.32 | 0.11 | -0.29 | 0.00 | 0.04 | -0.09 | -0.62*** |
| posteriorcingulate | 0.11 | 0.47* | 0.09 | 0.13 | 0.17 | -0.14 | -0.35 | 0.06 | 0.11 | 0.11 | 0.05 | 0.49* |

**Abbreviations:** GMV: grey matter volume; IRE: insular glioma related epilepsy; IRnE: insular tumor without epilepsy; IRE_L: IRE group with tumors in the left hemisphere; IRE_R: IRE group with tumors in the right hemispher; MO: the percentage of monocytes; HGB: hemoglobin; MCV: mean corpuscular volume; MCH: mean corpuscular hemoglobin; MCHC: mean corpuscular hemoglobin concentration; RDW: red cell distribution width; RDW-CV: red cell distribution width-coefficient of variation; PDW: platelet distribution width; MPV: mean platelet volume; P-LCR: platelet-large cell ratio; PCT: plateletcrit; HCT: hematocrit. The analysis relied on Spearman correlation analysis. Correlation > 0 indicated a potential positive association. Correlation < 0 indicated a potential negative association. *: *p* < 0.05; **: *p* < .01; ***: *p* < .001
