# Supplementary material for: The Dual Associations of Peripheral Inflammatory Cells With Brain Reorganization in Insular Gliomas With/Without Epilepsy: An Exploratory Analysis
Source: CNS Neurosci Ther. 2026 Feb 20;32(2):e70788. doi: 10.1002/cns.70788 (PMC12927981; doi:10.1002/cns.70788)
Supplement: Supplementary file 28 — Table S22: Regression analysis of potential biomarkers in peripheral blood. [file CNS-32-e70788-s024.docx]

**Table S22. Regression analysis of potential biomarkers in peripheral blood**

| **Group** | **Variables** | **Coef.**^a^ | **Std. err.** | ***t*** | **p > \|t\|** | **95% CI** |
| --- | --- | --- | --- | --- | --- | --- |
| IRE | WBC | -0.0022 | 0.001 | -3.196 | 0.002 | -0.004 to -0.001 |
|  | NEUT | -0.004 | 0.001 | -3.153 | 0.003 | -0.007 to -0.001 |
|  | MONO | -0.0254 | 0.011 | -2.387 | 0.02 | -0.047 to -0.004 |
| IRnE | WBC | 0.2112 | 0.078 | 2.702 | 0.008 | 0.056 to 0.366 |
|  | NEUT | 0.2577 | 0.089 | 2.882 | 0.006 | 0.078 to 0.437 |
|  | MONO | 2.0601 | 0.958 | 2.15 | 0.034 | 0.159 to 3.961 |

**Abbreviations:** IRE: insular glioma related epilepsy; IRnE: insular glioma without epilepsy; Coef.: Coefficient; Std. err.: standard error; *p*: *p* value; 95% CI: 95% confidence interval. WBC: white blood cell; MONO: monocyte; NEUT: neutrophil. The relatively small coefficients of WBC, NEUT and MONO for the IRE compensation (*Toro GI*) may be attributed to the inherently low values of *Toro GI* itself. The wide 95% CI of the coefficients for MONO in relation to the IRnE compensation (*GI*) is likely due to a limited sample size.
