# Supplementary material for: The Dual Associations of Peripheral Inflammatory Cells With Brain Reorganization in Insular Gliomas With/Without Epilepsy: An Exploratory Analysis
Source: CNS Neurosci Ther. 2026 Feb 20;32(2):e70788. doi: 10.1002/cns.70788 (PMC12927981; doi:10.1002/cns.70788)
Supplement: Supplementary file 29 — Table S23: Correlation matrix of the whole brain compensation regions and potential biomarkers in peripheral blood. [file CNS-32-e70788-s004.docx]

**Table S23. Correlation matrix of the whole brain compensation regions and potential biomarkers in peripheral blood**

| Peripheral blood components | Brain reorganization | | | |
| --- | --- | --- | --- | --- |
|  | Toro *GI* in IRE | | *GI* in IRnE | |
|  | Correlation | *p* | Correlation | *p* |
| RBC | 0.09 | 0.67 | -0.21 | 0.31 |
| LY | 0.21 | 0.31 | 0.09 | 0.66 |
| EO | -0.16 | 0.43 | -0.12 | 0.56 |
| BA | 0.04 | 0.86 | 0.25 | 0.23 |
| PLT | -0.09 | 0.68 | -0.09 | 0.68 |
| ALT | -0.20 | 0.35 | -0.12 | 0.58 |
| AST | 0.31 | 0.13 | -0.26 | 0.20 |
| TP | -0.07 | 0.73 | -0.01 | 0.95 |
| ALB | 0.13 | 0.53 | -0.08 | 0.69 |
| TBIL | -0.05 | 0.82 | -0.07 | 0.76 |
| DBIL | -0.03 | 0.90 | 0.04 | 0.87 |
| Urea | -0.19 | 0.37 | 0.11 | 0.61 |
| Cr | -0.23 | 0.28 | 0.16 | 0.43 |
| UA | 0.12 | 0.57 | 0.01 | 0.95 |
| GBL | -0.05 | 0.82 | 0.11 | 0.59 |
| A/G | 0.10 | 0.63 | -0.18 | 0.38 |
| IBIL | -0.04 | 0.84 | -0.08 | 0.70 |
| MO | 0.15 | 0.47 | 0.10 | 0.62 |
| GR | -0.14 | 0.50 | -0.04 | 0.85 |
| HGB | 0.09 | 0.67 | -0.12 | 0.57 |
| MCV | 0.11 | 0.59 | 0.15 | 0.49 |
| MCH | 0.11 | 0.60 | 0.22 | 0.29 |
| MCHC | -0.03 | 0.89 | -0.19 | 0.37 |
| RDW | -0.06 | 0.76 | 0.22 | 0.28 |
| RDW-CV | -0.07 | 0.74 | 0.19 | 0.35 |
| PDW | -0.07 | 0.72 | 0.22 | 0.28 |
| MPV | -0.14 | 0.51 | 0.26 | 0.20 |
| P-LCR | -0.20 | 0.34 | 0.26 | 0.21 |
| PCT | -0.22 | 0.30 | 0.00 | 1.00 |
| HCT | 0.18 | 0.39 | -0.15 | 0.47 |

**Abbreviations:** IRE: insular glioma related epilepsy; IRnE: insular tumor without epilepsy; *GI*: gyrification; Toro *GI*: toroidal *GI*; *p*: *p* value; RBC: red blood cell; LY: lymphocyte; EO: eosinophil; BA: basophil; PLT: platelet; ALT: alanine aminotransferase; AST: aspartate aminotransferase; TP: total protein; ALB: albumin; TBIL: total bilirubin; DBIL: direct bilirubin; Urea: blood urea nitrogen; Cr: creatinine; UA: uric acid; GBL: globulin; A/G: albumin/globulin ratio. IBIL: indirect bilirubin; MO: the percentage of monocytes; GR: the percentage of granulocytes; HGB: hemoglobin; MCV: mean corpuscular volume; MCH: mean corpuscular hemoglobin; MCHC: mean corpuscular hemoglobin concentration; RDW: red cell distribution width; RDW-CV: red cell distribution width-coefficient of variation; PDW: platelet distribution width; MPV: mean platelet volume; P-LCR: platelet large cell ratio; PCT: plateletcrit; HCT: hematocrit. The analysis relied on Spearman correlation analysis. Correlation > 0 indicated a potential positive association. Correlation < 0 indicated a potential negative association.
