# Supplementary material for: The Dual Associations of Peripheral Inflammatory Cells With Brain Reorganization in Insular Gliomas With/Without Epilepsy: An Exploratory Analysis
Source: CNS Neurosci Ther. 2026 Feb 20;32(2):e70788. doi: 10.1002/cns.70788 (PMC12927981; doi:10.1002/cns.70788)
Supplement: Supplementary file 30 — Table S24: Analysis of potential causal link between specific peripheral blood cells and brain reorganization. [file CNS-32-e70788-s010.docx]

**Table S24. The analysis of potential causal link between specific peripheral blood cells and brain reorganization.**

| **Model** | **Direction** | **Beta.** | **SE.** | **95% CI** | **R^2^** | **Adj. R^2^** | **AIC** | **BIC** | **Durbin**  **Watson** | **Residual**  **SD.** | ***p* value** |
| --- | --- | --- | --- | --- | --- | --- | --- | --- | --- | --- | --- |
| Model A | WBC to Brain reorganization | 0.1907 | 0.0678 | 0.0476-0.3338 | 0.9973 | 0.997 | 47.15 | 50.14 | 2.45 | 0.695 | 0.012 |
|  | NEUT to Brain reorganization | 0.0214 | 0.0079 | 0.0051-0.0377 | 0.286 | 0.247 | 112.38 | 121.52 | 1.95 | 0.181 | 0.0098 |
|  | MONO to Brain reorganization | 0.0187 | 0.0064 | 0.0058-0.0316 | 0.322 | 0.296 | 104.72 | 113.58 | 1.98 | 0.167 | 0.0052 |
| Model B | Brain reorganization to WBC | 1.6651 | 0.5921 | 0.416-2.9143 | 0.5044 | 0.4461 | 90.49 | 93.48 | 2.04 | 2.0523 | 0.012 |
|  | Brain reorganization to NEUT | 1.4732 | 0.5521 | 0.3568-2.5896 | 0.215 | 0.168 | 118.94 | 128.08 | 1.87 | 0.916 | 0.0116 |
|  | Brain reorganization to MONO | 1.2954 | 0.4839 | 0.3259-2.2649 | 0.244 | 0.21 | 111.49 | 120.35 | 1.91 | 0.742 | 0.0097 |

**Abbreviations:** Beta: coefficient; SE: standard error; CI: confidence interval; R²: coefficient of determination; Adj. R²: adjusted R²; AIC: Akaike information criterion; BIC: Bayesian information criterion; DW: Durbin-Watson statistic; SD: residual standard deviation. WBC: white blood cell; NEUT: neutrophil; MONO: monocyte. **The detailed causal link was not explained ensured the table was clear.** Model A meant that the special peripheral blood cells would affect the brain reorganization. Model B meant that the brain reorganization would affect the special peripheral blood cells.
